# Childhood adversity and cardiometabolic biomarkers in mid-adulthood in the 1958 British birth cohort

**DOI:** 10.1016/j.ssmph.2022.101260

**Published:** 2022-10-04

**Authors:** Natalie Ella Miller, Rebecca E. Lacey

**Affiliations:** Department of Epidemiology and Public Health, Institute of Epidemiology and Health Care, University College London, London, UK

**Keywords:** Childhood adversity, Cohort, Cardiovascular disease, Cardiometabolic risk

## Abstract

Studies that have examined associations between adverse childhood experiences (ACEs) and cardiometabolic biomarkers in adulthood are limited as they mainly focus on childhood maltreatment. This study aimed to examine the association between a range of prospectively and retrospectively reported ACEs and cardiometabolic biomarkers in mid-adulthood. Multiply-imputed data on 8511 participants from the National Child Development Study (1958 British birth cohort) were used. ACEs were prospectively reported at ages 7, 11 and 16, and retrospectively reported at age 33/44/45. Cardiometabolic outcomes assessed at age 44/45 included glycated haemoglobin (HbA1c), cholesterol (total, low-density lipoprotein (LDL) and high-density lipoprotein (HDL)), triglycerides, blood pressure (systolic and diastolic), body mass index, waist circumference and metabolic syndrome. Parental separation/divorce, physical neglect, emotional neglect and psychological abuse were associated with lower HDL cholesterol. Parental offending and physical neglect were associated with higher triglyceride concentrations. Parental offending was also associated with increased HbA1c. Exposure to 2+ (vs. 0) prospective ACEs was associated with lower HDL cholesterol. All these associations were after adjustment for sex and multiple early life factors. To conclude, several individual ACEs are associated with poorer cardiometabolic risk factor profiles in mid-adulthood. Furthermore, exposure to two or more prospective ACEs is associated with lower HDL cholesterol concentrations in mid-adulthood.

## Highlights

• Parental separation/divorce in childhood is associated with lower high-density lipoprotein cholesterol in mid-adulthood.
• Physical neglect in childhood is associated with poorer lipid profiles in mid-adulthood.
• Psychological abuse in childhood is associated with lower high-density lipoprotein cholesterol in mid-adulthood.
• Parental offending in childhood is associated with higher triglycerides and glycated haemoglobin in mid-adulthood.

## Abbreviations

ACE: adverse childhood experience
BMI: body mass index
CVD: cardiovascular disease
HbA1c: glycated haemoglobin
HDL: high-density lipoprotein
LDL: low-density lipoprotein
MDD: major depressive disorder
MI: multiple imputation

## 1 Introduction

Adverse childhood experiences (ACEs) are stressful early life experiences that require substantial psychological, social or neurobiological adaptation for an average child, and deviate from the typical, expected environment (McLaughlin, 2016). ACEs tend to co-occur, meaning that children who experience one adversity are more likely to experience another (Lacey et al., 2020a). Further, ACEs are highly prevalent in several major countries, with studies showing that approximately half of all individuals in the US and UK have experienced at least 1 ACE (Bellis et al., 2014a; Giano et al., 2020). Their high prevalence is particularly concerning given that ACEs have been linked with adverse health outcomes in later life (Hughes et al., 2017), such as cardiovascular disease (CVD) incidence and mortality (Basu et al., 2017; Pool et al., 2021; Su et al., 2015a; Suglia et al., 2015). Given the high prevalence of ACEs and their link with later adverse health outcomes, ACEs are a major public health issue.

Growing evidence also shows that ACEs are associated with several CVD risk factors (Danese & Tan, 2014; Gooding et al., 2015; Ágnes et al., 2019). Some potential mechanisms linking ACEs and CVD risk factors include allostatic load (McEwen, 2000), health risk behaviours (e.g., smoking, alcohol consumption, obesity) (Bellis et al., 2014b), low socioeconomic position (Misiak et al., 2022) and psychological processes (e.g., depression/anxiety) (Elderon & Whooley, 2013). For instance, many studies have found that childhood adversity is associated with higher triglyceride and low-density lipoprotein (LDL) cholesterol concentrations and lower high-density lipoprotein (HDL) and total cholesterol concentrations in adulthood (Kraav et al., 2019; Misiak et al., 2015; Ágnes et al., 2019). However, in all these studies childhood adversities were retrospectively reported in adulthood, which might be inaccurate due to social desirability and memory biases. Furthermore, all these studies were conducted in clinical populations such as adults with major depressive disorder (MDD) or schizophrenia. Thus, findings may not be generalisable to the general population. Studies conducted in general population participants have found associations between cumulative ACE exposure, finding that more adversities were associated with higher blood pressure (Su et al., 2015b), higher body mass index (BMI) and higher waist circumference (Pretty, O'Leary, Cairney, & Wade, 2013a, 2013b). However, these studies examined associations with few CVD risk factor outcomes which does not fully represent total CVD risk. Studies that have examined associations between cumulative ACE exposure and multiple CVD risk factors have only considered childhood maltreatment and thus associations between broader childhood adversities, such as parental mental health problems and parental separation, and CVD risk profiles remain unknown (Li et al., 2019). Furthermore, although cumulative ACE scores are valuable as ACEs tend to co-occur, these scores are also somewhat limited as they place equal weighting on all individual ACEs even though different ACEs might have different associations with health outcomes (Lacey & Minnis, 2020). Thus, it is helpful to consider both individual and cumulative ACE scores in complementary analyses to explore 1) which ACEs have effects on health and 2) the cumulative impact of ACEs on health.

Several population-based studies have examined associations between individual ACEs and CVD risk factors (Goncalves Soares et al., 2021; Gooding et al., 2014; Li et al., 2019; Suglia et al., 2014). However, studies have found inconsistent findings with regard to the association between child maltreatment and high blood pressure. Specifically, some studies show that child maltreatment is associated with higher blood pressure (Suglia et al., 2014) and others show no association between the two (Gooding et al., 2014). One reason for these inconsistent findings is that not all studies have prospective data so have been unable to control for early life factors such as socio-economic position and birth weight which are associated with CVD risk (Lynch & Smith, 2005). Furthermore, like with cumulative ACE scores, most studies examining associations between individual ACEs and CVD risk factors have focused on childhood maltreatment (i.e., abuse and neglect) (Goncalves Soares et al., 2021; Li et al., 2019). Thus, associations between other common types of ACEs such as parental mental illness or parental separation/divorce with CVD risk factors remain unknown. Finally, prior studies examining associations between ACEs and CVD risk factors have only examined either prospective or retrospective ACEs (Anderson et al., 2017; Li et al., 2019). However, prospective and retrospective measures of ACEs do not correlate very well and identify different groups of individuals (Baldwin et al., 2019). Retrospective ACE measures have been shown to be less related to objectively measured health outcomes compared to prospective ACE measures (Reuben et al., 2016). Prospective ACE measures may be prone to under-reporting by parents or teachers due to potential legal or social implications (Jakubowski et al., 2018). Thus, it is important to consider both prospectively and retrospectively reported ACEs to assess the impact of reporting on the findings and conclusions of a study.

Consequently, the main aim of the present study was to examine the association between a range of prospectively and retrospectively measured individual and cumulative ACEs with cardiometabolic markers in mid-adulthood in the 1958 National Childhood Development Study (NCDS). We included both individual ACEs and cumulative ACE scores, considered multiple cardiometabolic markers to better encapsulate total CVD risk, and accounted for multiple sociodemographic and early life covariates.

## 2 Methods

### 2.1 Data

The NCDS follows the lives of 17,415 individuals born during a single week of 1958 (98.2% of all births that week) in England, Scotland and Wales (Power & Elliott, 2006). Since the study started, 12 waves of data collection have taken place: at birth and at ages 7, 11, 16, 23, 33, 42, 44/45, 46, 55 and 62 years. Surveys have assessed health, education, employment, social relationships and socioeconomic factors. Ethical approval was obtained for each sweep from 2000 by the National Health Service (NHS) Research Ethics Committee, and all participants provided informed written consent. Follow-ups prior to 2000 took place before the multicentre research ethics committee (MREC) system was established, so only internal ethical review was conducted for these surveys. The follow-up at age 44/45 included a biomedical survey conducted on a subsample of participants (*n* = 9377; 77.9% of those targeted) in which blood samples were taken by nurses. A questionnaire on childhood adversity was also administered at age 44/45.

### 2.2 Measures

#### 2.2.1 ACEs

In the NCDS, ACEs were measured prospectively at ages 7, 11 and 16 years through reports by parents (usually the mother), health visitors or teachers, and retrospectively by cohort members using a confidential computer-based questionnaire (at ages 33 and 44/45). Prospectively reported ACEs included parental separation/divorce, parental substance misuse, parental conflict, parental death, parental mental health problems, physical neglect, and parental offending. Retrospectively reported ACEs included parental separation/divorce (asked at age 33), parental substance misuse, family conflict, witnessing abuse, parental mental health problems, sexual abuse, physical abuse, psychological abuse, and emotional neglect.

We also considered ACEs cumulatively as both prospective and retrospective ACE scores. Prospective ACE scores ranged from “0 ACEs” to “2+ ACEs” and retrospective ACE scores ranged from “0 ACEs” to “4+ ACEs”. These categorisations were chosen based on the distribution of cohort members in these variables, have been defined previously in studies using the NCDS (Lacey et al., 2020b), and are consistent with the wider ACE score literature (Bellis et al., 2017; Felitti et al., 1998).

#### 2.2.2 Outcomes

Cardiometabolic biomarkers were obtained at ages 44/45 using standardised procedures by a trained study nurse (Centre for Longitudinal Studies, 2020). Height (cm), weight (kg) and waist circumference (cm) were measured, and BMI was calculated using the formula weight/(height)^2^. HDL cholesterol, total cholesterol and triglycerides were measured from non-fasting blood samples using an Olympus model AU640 autoanalyser. LDL cholesterol was calculated from non-fasting blood samples using the Friedewald formula (Friedewald et al., 1972). Glycated haemoglobin (HbA1c) was measured on citrated whole blood by ion exchange high-performance liquid chromatography, using the Tosoh A1c 2.2 Glycohemoglobin Analyser HLC-723GHb. Systolic and diastolic blood pressure were measured three times while seated using an Omron 705CP automated digital oscillometric sphygmomanometer, and the third readings were used in this study (Lacey et al., 2017). According to the National Cholesterol Education Program (NCEP) Adult Treatment Panel III (ATP III) definition of metabolic syndrome, individuals were considered to have metabolic syndrome if they had three of the following: abdominal obesity (waist circumference ≥102 cm in men and ≥88 cm in women); hypertriglyceridemia (triglycerides ≥1.70 mm/L); low HDL cholesterol (<1.03 mm/L in men and <1.29 mm/L in women); high blood pressure (≥130/85 mmHg); and/or high fasting glucose (>6.10 mmol/L) (Third Report of the National, 2002). Nurses recorded information on prescribed medications: antidiabetic/glucose-lowering (*n* = 152; 1.79%), lipid-regulating (*n* = 128; 1.50%) and anti-hypertensive (*n* = 391; 4.59%).

#### 2.2.3 Covariates

Covariates were chosen from similar studies investigating ACEs and cardiometabolic markers (Li et al., 2019). Sex was included as a covariate as well as three indicators of socioeconomic circumstances during childhood: father's occupational social class reported prospectively at birth (or at 7y if missing) classified as I/II (professional/managerial), IIINM (skilled non-manual), IIIM (skilled manual) and IV/V (semi-unskilled manual, including no male head); maternal education level at birth (left school before/after minimum leaving age); and household overcrowding assessed at 7y (people per room - categorised as crowded if >1 person per room). Six indicators of health at birth were also included as covariates: birth weight (grams); gestational age (days); maternal age at birth (years); maternal smoking during pregnancy (no smoking, sometimes, often, heavy); breastfeeding duration (no, one month or less, more than one month); and mother's BMI before childbirth (kg/m^2^).

### 2.3 Statistical analyses

To reduce the bias caused by missing data, we used multiple imputation (MI) by chained equations to impute missing information on predictors, outcomes and covariates given recommendations to impute all three (Sterne et al., 2009). To make the missing at random assumption more plausible, we included auxiliary variables associated with non-response in the NCDS in imputation models: overcrowding assessed at 16 years; free school meal receipt assessed at 11 and 16 years; and financial hardship assessed at 11 and 16 years (Lacey et al., 2020b; Li et al., 2019). Twenty imputed datasets were generated. The imputed and observed data appeared to be similar, suggesting that the MI procedure worked. Analysis samples were restricted to individuals with data on all retrospectively reported ACEs at age 44/45 and at least one cardiometabolic biomarker (*N* = 8511).

To correct for medications, participants taking antidiabetic/glucose-lowering medication had their HbA1c levels corrected assuming that medication reduced HbA1c levels by 1% (Bennett et al., 2011); participants taking lipid-lowering drugs had their lipid levels corrected assuming that statins, the most commonly used lipid-lowering drug in this study, reduce total cholesterol by 20%, LDL cholesterol by 35% and triglycerides by 15%, and increase HDL cholesterol by 5% (Ki et al., 2011); and participants taking antihypertensive medication had their systolic blood pressure increased by 10 mmHg and diastolic blood pressure increased by 5 mmHg (Cui et al., 2003).

Triglycerides and HbA1c values were positively skewed so were log transformed prior to analyses and multiplied by 100 to assess % change in mean levels (Cole, 2000). Associations between ACEs (individual ACEs and ACE scores) with cardiometabolic outcomes adusting for all covariates were examined using linear regression for continuous and logistic regression for binary outcomes. Sensitivity analyses were conducted on participants with complete data on all variables included in each analysis prior to imputation (complete case analysis). As expected, the findings differed from the main analyses, probably because data were not missing completely at random, so the complete case analysis was biased (See Supplementary Tables 1-2). Additional sensitivity analyses examined the effects of 1) excluding individuals on medications and 2) adjusting rather than correcting for medications. The results from these analyses were similar to the main analyses and are shown in Supplementary Tables 3-6. Due to multiple testing, we applied Bonferroni corrections and used a corrected *p* value threshold of *p* ≤ 0.005 (0.05 divided by 10 outcomes for each exposure). For all analyses, we combined men and women as there was no evidence of differential associations between genders when a gender*ACE interaction term was added (*p* ≥ 0.005 for the interaction term). All analyses were conducted in Stata version 17.0 (StataCorp. Stata Statistical Software, 2021).

## 3 Results

### 3.1 Sample characteristics

The characteristics of the analytical sample (*N* = 8511) in both the observed and imputed data are shown in Table 1. With regards to the prospectively reported childhood adversities, the most commonly reported adversity in the imputed data was parental offending (6.34%), followed by parental separation/divorce (5.85%), parental mental illness (5.12%), physical neglect (4.91%), family conflict (4.56%), parental death (3.55%) and parental substance misuse (1.06%). When looking at prospective ACE scores, most individuals had no ACEs (78.24%), however 15.27% reported one ACE and 6.49% reported at least two ACEs. With regards to the retrospectively reported childhood adversities, the most commonly reported adversity was parental mental illness (25.26%), followed by parental substance misuse (13.11%), family conflict (12.29%), emotional neglect (10.99%), psychological abuse (8.92%), parental separation/divorce (8.11%), physical abuse (5.50%), witnessing abuse (5.37%) and sexual abuse (1.27%). When looking at ACE scores, most individuals retrospectively reported no ACEs (60.99%), however some individuals reported one ACE (16.36%), two ACEs (9.82%), three ACEs (4.88%) or at least four ACEs (7.96%).

**Table 1:** Characteristics of analytical sample (*N* = 8511)^a^.

	Observed		Imputed	
	Missing, N (%)	Median(IQR)/n(%)	Missing, N (%)	Median(IQR)/n(%)^b^
**Prospective childhood adversities (0–16 years)**				
Parental separation/divorce	2151 (25.27)		0 (0)	
Yes		331 (5.20)		498 (5.85)
No		6029 (94.80)		8013 (94.15)
Parental substance misuse	1973 (23.18)		0 (0)	
Yes		43 (0.66)		90 (1.06)
No		6495 (99.34)		8421 (98.94)
Parental death	2109 (24.78)		0 (0)	
Yes		217 (3.39)		302 (3.55)
No		6185 (96.61)		8209 (96.45)
Parental mental illness	1601 (18.81)		0 (0)	
Yes		348 (5.04)		436 (5.12)
No		6562 (94.96)		8075 (94.88)
Physical neglect	1055 (12.40)		0 (0)	
Yes		360 (4.83)		418 (4.91)
No		7096 (95.17)		8093 (95.09)
Parental offending	2510 (29.49)		0 (0)	
Yes		344 (5.73)		540 (6.34)
No		5657 (94.27)		7971 (93.66)
Family conflict	1966 (23.10)		0 (0)	
Yes		276 (4.22)		388 (4.56)
No		6269 (95.78)		8123 (95.44)
Prospective ACE score	3117 (36.62)		0 (0)	
0 ACEs		4395 (81.48)		6659 (78.24)
1 ACEs		728 (13.50)		1300 (15.27)
2+ ACEs		271 (5.02)		552 (6.49)
**Retrospective childhood adversities (33/44/45 years)**				
Parental separation/divorce	1353 (15.90)		0 (0)	
Yes		541 (7.56)		690 (8.11)
No		6617 (92.44)		7821 (91.89)
Parental substance misuse	0 (0)		0 (0)	
Yes		1116 (13.11)		1116 (13.11)
No		7395 (86.89)		7395 (86.89)
Parental mental illness	0 (0)		0 (0)	
Yes		2150 (25.26)		2150 (25.26)
No		6361 (74.74)		6361 (74.74)
Family conflict	0 (0)		0 (0)	
Yes		1046 (12.29)		1046 (12.29)
No		7465 (87.71)		7465 (87.71)
Emotional neglect	0 (0)		0 (0)	
Yes		935 (10.99)		935 (10.99)
No		7576 (89.01)		7576 (89.01)
Physical abuse	0 (0)		0 (0)	
Yes		468 (5.50)		468 (5.50)
No		8043 (94.50)		8043 (94.50)
Sexual abuse	0 (0)		0 (0)	
Yes		108 (1.27)		108 (1.27)
No		8403 (98.73)		8403 (98.73)
Psychological abuse	0 (0)		0 (0)	
Yes		759 (8.92)		759 (8.92)
No		7752 (91.08)		7752 (91.08)
Witnessed abuse	0 (0)		0 (0)	
Yes		457 (5.37)		457 (5.37)
No		8054 (94.63)		8054 (94.63)
Retrospective ACE score	1353 (15.90)		0 (0)	
0 ACEs		4500 (62.87)		5191 (60.99)
1 ACEs		1169 (16.33)		1392 (16.36)
2 ACEs		677 (9.46)		836 (9.82)
3 ACEs		306 (4.27)		415 (4.88)
4+ ACEs		506 (7.07)		677 (7.96)
**Cardiometabolic markers (44/45 years)**				
HbA1c (%)^c^	1213 (14.25)	5.2 (0.4)	0 (0)	5.2 (0.4)
Antidiabetic medication	0 (0)	152 (1.79)	0 (0)	152 (1.79)
HbA1c (%)^d^	1213 (14.25)	5.2 (0.4)	0 (0)	5.2 (0.4)
LDL cholesterol (mmol/L)^c^	1696 (19.93)	3.3 (1.2)	0 (0)	3.3 (1.2)
HDL cholesterol (mmol/L)^c^	1319 (15.50)	1.5 (0.5)	0 (0)	1.5 (0.5)
Total cholesterol (mmol/L)^c^	1306 (15.34)	5.8 (1.4)	0 (0)	5.8 (1.4)
Triglycerides (mmol/L)^c^	1330 (15.63)	1.6 (1.4)	0 (0)	1.7 (1.4)
Lipid-regulating medication	0 (0)	128 (1.50)	0 (0)	128 (1.50)
LDL cholesterol (mmol/L)^d^	1696 (19.93)	3.4 (1.2)	0 (0)	3.3 (1.2)
HDL cholesterol (mmol/L)^d^	1319 (15.50)	1.5 (0.5)	0 (0)	1.5 (0.5)
Total cholesterol (mmol/L)^d^	1306 (15.34)	5.8 (1.3)	0 (0)	5.8 (1.4)
Triglycerides (mmol/L)^d^	1330 (15.63)	1.6 (1.4)	0 (0)	1.7 (1.4)
Systolic blood pressure (mmHg)^c^	79 (0.93)	124 (21)	0 (0)	125 (21)
Diastolic blood pressure (mmHg)^c^	80 (0.94)	77 (15)	0 (0)	77.3 (15)
Antihypertensive medication	0 (0)	391 (4.59)	0 (0)	391 (4.59)
Systolic blood pressure (mmHg)^d^	79 (0.93)	124 (21)	0 (0)	124 (21)
Diastolic blood pressure (mmHg)^d^	80 (0.94)	78 (15)	0 (0)	78 (15)
BMI (kg/m^2^)	1271 (14.93)	26.72 (5.9)	0 (0)	26.84 (6.1)
Waist circumference (cm)	33 (0.39)	91.8 (18.6)	0 (0)	91.8 (18.6)
Metabolic syndrome	1506 (17.69)	943 (13.46)	0 (0)	1235 (14.51)
**Covariates**				
Gender, female	0 (0)	4276 (50.24)	0 (0)	4276 (50.24)
Father's social class (birth)	762 (8.95)		0 (0)	
I professional		403 (5.20)		439 (5.16)
II managerial & technical		1126 (14.53)		1237 (14.53)
IIINM skilled non-manual		801 (10.34)		881 (10.35)
IIIM skilled manual		3898 (50.30)		4279 (50.28)
IV semi-skilled manual		918 (11.85)		1007 (11.83)
V unskilled manual		603 (7.78)		669 (7.86)
Mother's education (birth)	456 (5.36)		0 (0)	
Stayed at school beyond minimum leaving age		2205 (27.37)		2340 (27.49)
Left school before minimum leaving age		5850 (72.63)		6171 (72.51)
Household overcrowding (7 years)	901 (10.59)		0 (0)	
Crowded (>1 person per room)		901 (12.45)		1077 (12.66)
Uncrowded (≤1 person per room)		6336 (87.55)		7434 (87.34)
Birth weight (grams)	701 (8.24)	3345.24 (652.0)	0 (0)	3345.24 (647.8)
Maternal age at birth (years)	438 (5.15)	27 (8)	0 (0)	27 (8)
Gestational age (days)	1181 (13.88)	282 (13)	0 (0)	282 (13.3)
Breastfeeding (reported at 7 years)	1050 (12.34)		0 (0)	
No		2171 (29.10)		2477 (29.10)
Up to 1 month		1805 (24.19)		2055 (24.15)
Longer than 1 month		3485 (46.71)		3979 (46.75)
Maternal smoking during pregnancy	528 (6.20)		0 (0)	
No		5466 (68.47)		5825 (68.44)
Yes		2517 (31.53)		2686 (31.56)
Mother's BMI before childbirth (kg/m^2^)	1321 (15.52)	23.06 (4.4)	0 (0)	23.06 (4.4)

*Note.* ACE, adverse childhood experience; BMI, body mass index; LDL, low-density lipoprotein; HDL, high-density lipoprotein.

a Sample consists of participants with complete data on retrospectively reported ACEs at age 44/45 and data on at least one observed cardiometabolic outcome.

b For imputed data, pooled *n*s and %s are shown across the 20 imputed datasets.

c Values not corrected for medications.

d Values corrected for medications.

### 3.2 Prospectively reported ACEs and cardiometabolic markers

Under Bonferroni correction, parental separation/divorce was associated with lower levels of HDL cholesterol after adjustment for sex and multiple early life covariates (*B* = −0.06; 95% CI: −0.10, −0.02) (Table 2). Parental offending was also associated with increased HbA1c (*B* = 1.98; 95% CI: 0.71, 3.26) and raised triglyceride levels after adjustment for the same covariates (*B* = 6.09; 95% CI: 2.47, 9.70). Physical neglect was associated with lower HDL cholesterol (*B* = −0.07; 95% CI: −0.11, −0.03) and raised triglycerides after multivariable adjustment (*B* = 7.68; 95% CI: 3.41, 11.94).

**Table 2:** Regression coefficients (mean difference, % difference or OR) showing associations between ACEs and cardiometabolic markers at age 44/45 in the NCDS.

	Prospective childhood adversity		Retrospective childhood adversity	
	*B* (95% CI)^c^	*p*	*B* (95% CI)^c^	*p*
Parental separation/divorce				
HbA1c^a^	0.74 (−0.45, 1.92)	0.225	−0.14 (−1.21, 0.92)	0.788
LDL cholesterol	0.02 (−0.08, 0.11)	0.701	−0.03 (−0.11, 0.06)	0.534
HDL cholesterol	**−0.06 (-0.10, -0.02)**	0.002*	**−0.05 (-0.08, -0.02)**	0.003*
Total cholesterol	0.02 (−0.09, 0.13)	0.759	−0.06 (−0.15, 0.04)	0.231
Triglycerides^a^	3.51 (−0.50, 7.52)	0.085	1.28 (−2.03, 4.59)	0.448
Systolic blood pressure	0.44 (−1.09, 1.97)	0.576	−0.33 (−1.82, 1.15)	0.659
Diastolic blood pressure	0.41 (−0.64, 1.46)	0.445	−0.06 (−1.04, 0.93)	0.912
BMI	0.40 (−0.09, 0.88)	0.108	0.09 (−0.32, 0.51)	0.665
Waist circumference	0.80 (−0.33, 1.93)	0.167	0.30 (−0.71, 1.30)	0.564
Metabolic syndrome^b^	1.21 (0.92, 1.58)	0.177	1.05 (0.81, 1.35)	0.722
Parental substance misuse				
HbA1c^a^	1.39 (−3.46, 6.24)	0.564	−0.27 (−1.04, 0.51)	0.497
LDL cholesterol	0.08 (−0.18, 0.35)	0.530	−0.03 (−0.10, 0.04)	0.353
HDL cholesterol	−0.01 (−0.12, 0.11)	0.898	−0.01 (−0.04, 0.02)	0.516
Total cholesterol	0.19 (−0.13, 0.52)	0.238	−0.04 (−0.11, 0.04)	0.348
Triglycerides^a^	5.78 (−4.79, 16.35)	0.280	0.40 (−2.06, 2.86)	0.749
Systolic blood pressure	0.82 (−3.88, 5.53)	0.728	−0.29 (−1.29, 0.71)	0.565
Diastolic blood pressure	0.23 (−2.58, 3.04)	0.872	−0.09 (−0.77, 0.59)	0.796
BMI	−0.13 (−1.45, 1.20)	0.851	0.13 (−0.19, 0.44)	0.425
Waist circumference	0.40 (−2.80, 3.59)	0.806	0.58 (−0.18, 1.33)	0.133
Metabolic syndrome^b^	1.21 (0.59, 2.45)	0.602	1.09 (0.90, 1.32)	0.377
Parental mental illness				
HbA1c^a^	0.35 (−0.88, 1.57)	0.579	−0.56 (−1.15, 0.02)	0.058
LDL cholesterol	0.05 (−0.06, 0.16)	0.371	0.00 (−0.05, 0.06)	0.857
HDL cholesterol	0.03 (−0.01, 0.07)	0.166	0.01 (−0.01, 0.03)	0.351
Total cholesterol	0.06 (−0.06, 0.18)	0.342	0.01 (−0.05, 0.07)	0.785
Triglycerides^a^	−2.13 (−5.87, 1.60)	0.263	−0.67 (−2.58, 1.23)	0.489
Systolic blood pressure	−0.54 (−2.14, 1.07)	0.512	−0.03 (−0.81, 0.74)	0.936
Diastolic blood pressure	0.03 (−1.08, 1.15)	0.954	0.19 (−0.34, 0.72)	0.475
BMI	−0.07 (−0.57, 0.43)	0.778	−0.13 (−0.37, 0.12)	0.299
Waist circumference	−0.21 (−1.41, 0.99)	0.732	−0.04 (−0.62, 0.55)	0.905
Metabolic syndrome^b^	0.94 (0.70, 1.27)	0.703	0.98 (0.84, 1.13)	0.739
Family conflict				
HbA1c^a^	0.61 (−1.04, 2.26)	0.467	0.19 (−0.60, 0.98)	0.636
LDL cholesterol	0.06 (−0.06, 0.18)	0.316	0.03 (−0.04, 0.10)	0.394
HDL cholesterol	−0.04 (−0.09, 0.00)	0.077	**−0.03 (-0.06, -0.002)**	**0.038**
Total cholesterol	0.06 (−0.08, 0.19)	0.412	0.01 (−0.07, 0.09)	0.774
Triglycerides^a^	2.13 (−2.34, 6.60)	0.348	0.88 (−1.63, 3.39)	0.491
Systolic blood pressure	−0.85 (−2.73, 1.02)	0.372	−1.73 (-2.75, -0.71)*	0.001*
Diastolic blood pressure	−0.01 (−1.27, 1.25)	0.990	**−0.75 (-1.45, -0.04)**	**0.038**
BMI	0.27 (−0.35, 0.90)	0.386	0.16 (−0.17, 0.49)	0.345
Waist circumference	0.96 (−0.50, 2.42)	0.197	0.49 (−0.28, 1.26)	0.216
Metabolic syndrome^b^	1.04 (0.74, 1.46)	0.809	1.13 (0.93, 1.37)	0.207
Witnessed abuse				
HbA1c^a^	–		−0.27 (−1.45, 0.91)	0.656
LDL cholesterol	–		**0.10 (0.002, 0.20)**	**0.045**
HDL cholesterol	–		−0.02 (−0.06, 0.02)	0.291
Total cholesterol	–		0.10 (−0.01, 0.21)	0.085
Triglycerides^a^	–		2.54 (−1.25, 6.32)	0.189
Systolic blood pressure	–		−0.91 (−2.41, 0.59)	0.236
Diastolic blood pressure	–		0.06 (−0.96, 1.09)	0.904
BMI	–		0.40 (−0.07, 0.86)	0.097
Waist circumference	–		**1.18 (0.06, 2.31)**	**0.039**
Metabolic syndrome^b^	–		1.11 (0.84, 1.48)	0.463
Parental death				
HbA1c^a^	0.33 (−1.14, 1.81)	0.658	–	
LDL cholesterol	0.04 (−0.09, 0.17)	0.531	–	
HDL cholesterol	−0.04 (−0.10, 0.01)	0.103	–	
Total cholesterol	−0.05 (−0.20, 0.10)	0.514	–	
Triglycerides^a^	−2.36 (−7.32, 2.59)	0.348	–	
Systolic blood pressure	−0.78 (−2.74, 1.19)	0.437	–	
Diastolic blood pressure	−0.16 (−1.52, 1.20)	0.817	–	
BMI	0.38 (−0.24, 1.00)	0.230	–	
Waist circumference	1.02 (−0.48, 2.51)	0.183	–	
Metabolic syndrome^b^	1.24 (0.88, 1.75)	0.228	–	
Parental offending				
HbA1c^a^	**1.98 (0.71, 3.26)**	0.003*	–	
LDL cholesterol	−0.03 (−0.12, 0.07)	0.589	–	
HDL cholesterol	−0.03 (−0.07, 0.01)	0.138	–	
Total cholesterol	0.05 (−0.06, 0.16)	0.348	–	
Triglycerides^a^	**6.09 (2.47, 9.70)**	0.001*	–	
Systolic blood pressure	0.78 (−0.80, 2.36)	0.333	–	
Diastolic blood pressure	0.66 (−0.39, 1.71)	0.221	–	
BMI	0.37 (−0.12, 0.86)	0.137	–	
Waist circumference	1.15 (−0.02, 2.33)	0.053	–	
Metabolic syndrome^b^	**1.39 (1.08, 1.80)**	**0.011**	–	
Physical neglect				
HbA1c^a^	0.98 (−0.43, 2.40)	0.172	–	
LDL cholesterol	0.01 (−0.09, 0.12)	0.795	–	
HDL cholesterol	**−0.07 (-0.11, -0.03)**	0.001*	–	
Total cholesterol	0.07 (−0.05, 0.19)	0.269	–	
Triglycerides^a^	**7.68 (3.41, 11.94)**	< 0.001*	–	
Systolic blood pressure	−0.52 (−2.20, 1.17)	0.546	–	
Diastolic blood pressure	−0.31 (−1.43, 0.82)	0.594	–	
BMI	**−0.57 (-1.11, -0.03)**	**0.038**	–	
Waist circumference	−0.78 (−2.04, 0.48)	0.224	–	
Metabolic syndrome^b^	1.03 (0.77, 1.38)	0.840	–	
Emotional neglect				
HbA1c^a^	–		0.10 (−0.71, 0.92)	0.801
LDL cholesterol	–		0.02 (−0.05, 0.09)	0.551
HDL cholesterol	–		**−0.05 (-0.08, -0.02)**	0.001*
Total cholesterol	–		−0.01 (−0.09, 0.07)	0.766
Triglycerides^a^	–		1.56 (−1.06, 4.18)	0.243
Systolic blood pressure	–		**−1.65 (-2.72, -0.58)**	0.003*
Diastolic blood pressure	–		−0.52 (−1.26, 0.21)	0.164
BMI	–		−0.16 (−0.50, 0.17)	0.343
Waist circumference	–		−0.40, (−1.21, 0.40)	0.329
Metabolic syndrome^b^	–		1.12 (0.92, 1.37)	0.254
Physical abuse				
HbA1c^a^	–		0.59 (−0.64, 1.83)	0.347
LDL cholesterol	–		**0.11 (0.01, 0.21)**	**0.032**
HDL cholesterol	–		−0.03 (−0.07, 0.01)	0.174
Total cholesterol	–		0.10 (−0.01, 0.22)	0.085
Triglycerides^a^	–		2.06 (−1.66, 5.78)	0.277
Systolic blood pressure	–		−0.82 (−2.30, 0.66)	0.278
Diastolic blood pressure	–		−0.38 (−1.39, 0.63)	0.457
BMI	–		**0.54 (0.08, 1.00)**	**0.022**
Waist circumference	–		1.01 (−0.10, 2.12)	0.074
Metabolic syndrome^b^	–		1.14 (0.86, 1.49)	0.362
Sexual abuse				
HbA1c^a^	–		−0.10 (−2.38, 2.18)	0.931
LDL cholesterol	–		0.10 (−0.09, 0.29)	0.306
HDL cholesterol	–		−0.02 (−0.10, 0.05)	0.571
Total cholesterol	–		0.01 (−0.21, 0.23)	0.926
Triglycerides^a^	–		−3.98 (−11.41, 3.46)	0.294
Systolic blood pressure	–		−2.89 (−5.94, 0.17)	0.064
Diastolic blood pressure	–		−1.92 (−4.02, 0.17)	0.072
BMI	–		−0.11 (−1.08, 0.86)	0.826
Waist circumference	–		−0.95 (−3.23, 1.33)	0.413
Metabolic syndrome^b^	–		0.93 (0.50, 1.70)	0.806
Psychological abuse				
HbA1c^a^	–		0.07 (−0.88, 1.02)	0.890
LDL cholesterol	–		0.07 (−0.01, 0.15)	0.103
HDL cholesterol	–		−0.05 (-0.08, -0.02)*	0.003*
Total cholesterol	–		0.02 (−0.07, 0.12)	0.614
Triglycerides^a^	–		0.99 (−1.99, 3.96)	0.514
Systolic blood pressure	–		−0.61 (−1.79, 0.58)	0.313
Diastolic blood pressure	–		0.10 (−0.71, 0.91)	0.802
BMI	–		0.35 (−0.02, 0.73)	0.065
Waist circumference	–		**0.92 (0.03, 1.80)**	**0.043**
Metabolic syndrome^b^	–		1.12 (0.90, 1.39)	0.302

*Note.* Associations with *p ≤* 0.05 are presented in bold-face.

ACE, adverse childhood experience; BMI, body mass index; LDL, low-density lipoprotein; HDL, high-density lipoprotein; OR, odds ratio.

*Findings significant under Bonferroni correction (*p ≤* 0.005).

a Results presented as % differences (95% CI) as outcomes were positively skewed and log transformed prior to analysis.

b Results presented as ORs (95% CI).

c Models are adjusted for sex, father's occupation at birth, maternal education level at birth, household overcrowding at 7 years, birth weight, gestational age, maternal age at birth, maternal smoking during pregnancy and mother's BMI before childbirth.

### 3.3 Retrospectively reported ACEs and cardiometabolic markers

Under Bonferroni correction, parental separation/divorce was associated with lower HDL cholesterol after adjustment for sex and early life factors (*B* = −0.05; 95% CI -0.08, −0.02). Family conflict was associated with lower systolic blood pressure after multivariable adjustment (*B* = −1.73; 95% CI: −2.75, −0.71). Emotional neglect was associated with lower HDL cholesterol (*B* = −0.05; 95% CI: −0.08, −0.02) and lower systolic blood pressure after adjustment for sex and early life covariates (*B* = −1.65; 95% CI: −2.72, −0.58). Psychological abuse was associated with lower HDL cholesterol after adjustment for all covariates (*B* = −0.05; 95% CI: −0.08, −0.02).

### 3.4 Cumulative ACE scores and cardiometabolic markers

A prospective ACE score of 2+ (vs. 0) was associated with lower HDL cholesterol after adjustment for sex and multiple early life covariates (*B* = −0.06, 95% CI: −0.10, −0.02) (Table 3). All other associations between retrospective and prospective cumulative ACE scores with cardiometabolic markers were not statistically significant after multivariable adjustment under Bonferroni correction.

**Table 3:** Regression coefficients (mean difference, % difference or OR) showing associations between ACE scores and cardiometabolic markers at age 44/45 in the NCDS.

	Prospective childhood adversity			Retrospective childhood adversity		
		*B* (95% CI)^c^	*p*		*B* (95% CI)^c^	*p*
HbA1c^a^						
	0 ACEs	Ref		0 ACEs	Ref	
	1 ACEs	0.26 (−0.52, 1.04)	0.510	1 ACEs	−0.26 (−0.97, 0.44)	0.466
	2+ ACEs	**1.38 (0.18, 2.59)**	**0.025**	2 ACEs	−0.85 (−1.72, 0.02)	0.055
				3 ACEs	−0.48 (−1.70, 0.74)	0.442
				4+ ACEs	0.15 (−0.86, 1.16)	0.767
LDL cholesterol						
	0 ACEs	Ref		0 ACEs	Ref	
	1 ACEs	**0.08 (0.01, 0.14)**	**0.023**	1 ACEs	0.01 (−0.06, 0.07)	0.833
	2+ ACEs	0.00 (−0.10, 0.09)	0.947	2 ACEs	0.00 (−0.08, 0.08)	0.968
				3 ACEs	−0.05 (−0.16, 0.05)	0.309
				4+ ACEs	0.05 (−0.04, 0.13)	0.258
HDL cholesterol						
	0 ACEs	Ref		0 ACEs	Ref	
	1 ACEs	−0.02 (−0.05, 0.00)	0.069	1 ACEs	−0.02 (−0.04, 0.01)	0.192
	2+ ACEs	**−0.06 (-0.10, -0.02)**	0.002*	2 ACEs	0.00 (−0.03, 0.03)	0.978
				3 ACEs	−0.02 (−0.07, 0.02)	0.266
				4+ ACEs	**−0.04 (-0.07, -0.004)**	**0.029**
Total cholesterol						
	0 ACEs	Ref		0 ACEs	Ref	
	1 ACEs	**0.08 (0.01, 0.15)**	**0.031**	1 ACEs	0.01 (−0.06, 0.08)	0.715
	2+ ACEs	0.03 (−0.08, 0.14)	0.606	2 ACEs	0.00 (−0.09, 0.09)	0.980
				3 ACEs	−0.09 (−0.21, 0.03)	0.140
				4+ ACEs	0.02 (−0.08, 0.12)	0.717
Triglycerides^a^						
	0 ACEs	Ref		0 ACEs	Ref	
	1 ACEs	1.99 (−0.44, 4.42)	0.108	1 ACEs	1.37 (−0.98, 3.72)	0.253
	2+ ACEs	**4.63 (0.69, 8.57)**	**0.021**	2 ACEs	0.48 (−2.33, 3.29)	0.737
				3 ACEs	−0.94 (−4.91, 3.03)	0.641
				4+ ACEs	0.97 (−2.12, 4.07)	0.538
Systolic blood pressure						
	0 ACEs	Ref		0 ACEs	Ref	
	1 ACEs	−0.05 (−1.10, 0.99)	0.918	1 ACEs	0.06 (−0.88, 1.00)	0.900
	2+ ACEs	0.14 (−1.40, 1.68)	0.859	2 ACEs	−0.50 (−1.66, 0.67)	0.405
				3 ACEs	**−1.98 (-3.60, -0.36)**	**0.017**
				4+ ACEs	−0.80 (−2.09, 0.49)	0.226
Diastolic blood pressure						
	0 ACEs	Ref		0 ACEs	Ref	
	1 ACEs	0.09 (−0.62, 0.81)	0.798	1 ACEs	0.20 (−0.45, 0.85)	0.550
	2+ ACEs	0.37 (−0.67, 1.41)	0.487	2 ACEs	0.07 (−0.72, 0.87)	0.855
				3 ACEs	−1.06 (−2.17, 0.06)	0.063
				4+ ACEs	−0.08 (−0.96, 0.80)	0.855
BMI						
	0 ACEs	Ref		0 ACEs	Ref	
	1 ACEs	−0.06 (−0.38, 0.26)	0.724	1 ACEs	−0.11 (−0.40, 0.19)	0.470
	2+ ACEs	0.29 (−0.17, 0.75)	0.210	2 ACEs	−0.01 (−0.37, 0.36)	0.977
				3 ACEs	−0.21 (−0.71, 0.29)	0.404
				4+ ACEs	0.20 (−0.21, 0.60)	0.340
Waist circumference						
	0 ACEs	Ref		0 ACEs	Ref	
	1 ACEs	0.12 (−0.65, 0.88)	0.762	1 ACEs	−0.11 (−0.82, 0.59)	0.754
	2+ ACEs	0.91 (−0.24, 2.05)	0.120	2 ACEs	0.42 (−0.46, 1.29)	0.352
				3 ACEs	−0.18 (−1.39, 1.02)	0.765
				4+ ACEs	0.50 (−0.46, 1.46)	0.310
Metabolic syndrome^b^						
	0 ACEs	Ref		0 ACEs	Ref	
	1 ACEs	1.01 (0.83, 1.22)	0.931	1 ACEs	1.12 (0.94, 1.34)	0.200
	2+ ACEs	**1.31 (1.01, 1.68)**	**0.038**	2 ACEs	0.98 (0.78, 1.23)	0.866
				3 ACEs	1.01 (0.74, 1.37)	0.969
				4+ ACEs	1.18 (0.92, 1.51)	0.182

*Note.* Associations with *p ≤* 0.05 are presented in bold-face.

*Findings significant under Bonferroni correction (*p ≤* 0.005).

ACE, adverse childhood experience; BMI, body mass index; LDL, low-density lipoprotein; HDL, high-density lipoprotein; OR, odds ratio.

a Results presented as % differences (95% CI) as outcomes were positively skewed and log transformed prior to analysis.

b Results presented as ORs (95% CI).

c Models are adjusted for sex, father's occupation at birth, maternal education level at birth, household overcrowding at 7 years, birth weight, gestational age, maternal age at birth, maternal smoking during pregnancy and mother's BMI before childbirth.

## 4 Discussion

To conclude, the present study showed that several ACEs, namely parental separation/divorce, parental offending, physical neglect, emotional neglect and psychological abuse, were associated with poorer cardiometabolic risk factor profiles in mid-adulthood. These associations were independent of multiple early life factors including birth weight and socioeconomic position. Interestingly, the present study also found that retrospectively reported family conflict and emotional neglect were associated with lower systolic blood pressure in mid-adulthood, independently of sex and several early life factors. Prospectively reported family conflict, however, was not associated with blood pressure after multivariable adjustment. Finally, this study found that exposure to two or more prospectively reported ACEs compared to none was associated with lower levels of HDL cholesterol. No other associations between cumulative prospective or retrospective ACE scores and cardiometabolic risk factors in mid-adulthood were found.

A large body of evidence has shown that specific types of ACEs, particularly neglect and abuse, are associated with cardiometabolic risk factors in later life (Li et al., 2019; Spann et al., 2014; Ágnes et al., 2019). For instance, a study of 452 low SES African Americans found that child abuse was associated with lower HDL cholesterol concentrations in adulthood (Spann et al., 2014). Furthermore, a population-based study of approximately 9000 individuals from the NCDS found that neglect was associated with raised triglyceride and HbA1c levels, and psychological abuse was associated with lower HDL cholesterol levels, independently of many early life covariates (Li et al., 2019). Our findings are in line with these studies and has also shown associations between non-maltreatment related ACEs, such as parental offending and parental separation/divorce, with poorer lipid profiles and greater HbA1c levels in mid-adulthood. However, unlike prior research, we did not find any differential associations between ACEs and cardiometabolic biomarkers between genders (Li et al., 2019). Gender differences in exposure to ACEs (Haahr-Pedersen et al., 2020), CVD prevalence (Peters et al., 2019) and associations between ACEs and CVD have been shown to exist (Soares et al., 2020). One potential reason for our lack of finding of differential associations between ACEs and cardiometabolic biomarkers between genders is that our use of Bonferroni correction for multiple testing was too conservative and led to a Type II error. Alternatively, it is possible that differential associations between genders found in prior research reflect a Type I error due to lack of adjustment for multiple testing (Li et al., 2019). Future research in other cohorts is needed to confirm whether differential associations between ACEs and cardiometabolic biomarkers between genders do exist.

Furthermore, we did not find any associations between individual ACEs and measures of adiposity such as BMI or waist circumference, unlike several prior studies (Boynton-Jarrett et al., 2012; Hemmingsson et al., 2014; Hughes et al., 2017). One reason for our lack of associations is that unlike prior studies, we statistically controlled for maternal BMI before childbirth, which is causally associated with offspring adiposity (Noll et al., 2007; Richmond et al., 2017). We also controlled for early life factors which are associated with CVD risk in later life such as childhood socioeconomic position and birth weight, which prior studies have tended not to do (Lynch & Smith, 2005; Noll et al., 2007). Additionally, some studies that have found associations between child maltreatment and BMI have relied on self-report measures of BMI and have rarely extended beyond young adulthood (Shin & Miller, 2012). Thus, our findings suggest that certain individual ACEs may contribute to the development of CVD in later life through alterations in lipid profiles and HbA1c levels, rather than changes in adiposity.

There are several possible mechanisms underlying associations between specific ACEs and cardiometabolic biomarkers in mid-adulthood. One possible pathway is lifestyle behaviours. Studies have shown that exposure to ACEs is associated with greater odds of smoking, heavy alcohol consumption and obesity (Bellis et al., 2014b). Furthermore, studies examining associations between child abuse and CVD have found that lifestyle and medical risk factors are important mediators in this association (Rich-Edwards et al., 2012). Studies have also shown that behavioural factors (particularly smoking and alcohol consumption) mediate much of the association between childhood maltreatment and cardiometabolic biomarkers (Li et al., 2019). However, limited prior research has examined the role of behavioural factors in associations between non-maltreatment related ACEs and cardiometabolic biomarkers. Alternatively, associations between ACEs and cardiometabolic markers might be mediated by psychological processes. A sequential causal mediation analysis of data from the UK Biobank found that mental health (specifically depression/anxiety) mediated part of the association between childhood maltreatment and CVD (Soares et al., 2021). Depression/anxiety might also contribute to CVD risk through adverse health behaviours (Elderon & Whooley, 2013), suggesting that a complex pathway might exist between ACEs, depression/anxiety, health behaviours, cardiometabolic markers and CVD. Consequently, future work is needed to examine the roles of and interactions between mental health and health behaviours in the association between non-maltreatment related ACEs, cardiometabolic markers and CVD. Such work is important as it could lead to tailored interventions to reduce and prevent poor cardiometabolic health resulting from ACEs.

Interestingly, we also found that retrospectively reported family conflict and emotional neglect were associated with lower systolic blood pressure. Limited prior research has examined associations between family conflict and blood pressure in adulthood. However, a small-scale study of 39 African-American adolescents found that greater perceived family conflict predicted greater arterial blood pressure changes in adolescence (Clark & Armstead, 2000). Furthermore, a study of 122 female adolescents showed that negative social interactions were associated with a trajectory of increasing resting blood pressure over a two-year period (Ross et al., 2011). Several studies have examined associations between childhood maltreatment and blood pressure in adulthood, with some showing a positive association between the two (Su et al., 2015b) and others showing no association (Gooding et al., 2014; Li et al., 2019). One reason for our conflicting findings of a negative association between family conflict and emotional neglect with systolic blood pressure is that we corrected for blood pressure lowering medications whereas prior studies did not (Clark & Armstead, 2000; Gooding et al., 2014; Ross et al., 2011; Su et al., 2015b). Another possible reason is that studies examined associations between prospectively reported neglect and systolic blood pressure whereas in the present study, emotional neglect was retrospectively reported (Li et al., 2019). Retrospective reporting of ACEs is prone to underreporting due to the sensitive nature of ACEs as well as memory biases. Retrospective and prospective ACEs have been shown to be poorly correlated and tend to identify different groups of individuals (Baldwin et al., 2019). Evidence also suggests that retrospective reporting of ACEs might be influenced by personality traits (Reuben et al., 2016), mental health and life stress (Colman et al., 2016). For instance, increasing levels of psychological distress, work stress and chronic stress are associated with a greater likelihood of reporting a previously forgotten ACE (Colman et al., 2016). On the other hand, prospective ACE measures may be prone to under-reporting by parents or teachers, particularly if the perpetrator of the ACE is the reporter (i.e., emotional neglect by parent) (Jakubowski et al., 2018). In the present study we found no association between prospectively reported family conflict and blood pressure, suggesting that our finding of an association with retrospective family conflict/emotional neglect might well be biased by the mode of reporting. However, it is important to note that the measures used to capture ACEs retrospectively and prospectively in the NCDS were not the same, so direct comparisons cannot be made. Alternatively, it is possible that these associations reflect type 1 errors due to the large number of comparisons and statistical tests conducted in the present study. However, we did apply a Bonferroni correction to reduce the risk of a type 1 error resulting from multiple testing. Bonferroni correction can in fact be too conservative and lead to a high rate of false negatives as this correction assumes that all statistical tests are independent of each other (White et al., 2019). Nevertheless, our findings of an association between retrospective family conflict and emotional neglect with lower systolic blood pressure should still be interpreted with caution.

Exposure to two or more prospectively reported ACEs compared to none was associated with lower levels of HDL cholesterol in mid-adulthood. However, we found no other associations between cumulative prospective or retrospective ACE scores with cardiometabolic risk factors in mid-adulthood. The fact that no association was found between retrospective cumulative ACE scores and HDL cholesterol is probably because different ACEs were encapsulated in prospective and retrospective cumulative ACE scores. Accordingly, the association between two or more prospectively reported ACEs and lower HDL cholesterol was likely driven by associations between prospectively reported parental separation/divorce and physical neglect with lower HDL cholesterol. Several studies have investigated associations between cumulative childhood adversity with cardiometabolic risk factors in adulthood. A study of 2230 individuals from the MRC National Survey of Health and Development found no association between cumulative childhood adversity and HDL cholesterol, however, outcome assessment occurred at age 60–64 years (Baldwin et al., 2019). Given that our cohort were 44/45 years at outcome assessment, this suggests that associations between cumulative adversity and HDL cholesterol might attenuate with age. Other studies that have found associations between cumulative childhood adversity and cardiometabolic risk factors (e.g., BMI, triglycerides) are limited as they have either only explored a small number of adversities such as those encompassing maltreatment (Li et al., 2019) or have assessed outcomes in adolescence not adulthood (Pretty et al., 2013a, 2013b). Nevertheless, it is important to note that our study might not have been powered enough to detect many associations between cumulative ACE scores and cardiometabolic risk factors given the small proportion of individuals who had reported experiencing at least one ACE.

There are several notable strengths of the present study. First, the cohort were followed up for multiple decades. Furthermore, we included many different ACEs, early life covariates and cardiometabolic risk factors meaning that we were able to encompass total cardiometabolic disease risk and pinpoint associations between specific ACEs and cardiometabolic risk factors. In addition, we included both prospectively and retrospectively reported ACEs where possible which is a strength given that prospective and retrospective ACE measures tend to identify different groups of individuals (Baldwin et al., 2019). Therefore, we were able to test the sensitivity of the findings to both types of reporting. However, there are also some limitations of the present study that must be considered. Not all ACEs were measured both prospectively and retrospectively in the 1958 NCDS, which means that we cannot compare findings for all ACEs depending on the method of reporting. In addition, the observational design of this study means that causality cannot be concluded from associations, and there is potential for residual confounding by unmeasured factors. Another limitation of this study is the potential for bias caused by missing data, which is a common problem in cohort studies. Nevertheless, we used multiple imputation to impute missing values and included a wide range of predictor variables including some auxiliary variables to make the missing at random assumption more plausible. Thus, our use of multiple imputation aimed to reduce the bias and loss of power associated with attrition and non-response.

Consequently, the present study has shown that a wide spectrum of ACEs is associated with cardiometabolic risk factors in mid-adulthood, which may partly explain how ACEs contribute to CVD. Furthermore, exposure to two or more prospectively reported ACEs compared to none was associated with lower levels of HDL cholesterol in mid-adulthood. Our findings reinforce the need to develop strategies for preventing ACEs and the negative health consequences that may occur as a result.

## Ethical statement

Ethical approval was obtained for each sweep of the NCDS from 2000 by the National Health Service (NHS) Research Ethics Committee, and all participants provided informed written consent.

## Author statement

NM: Conceptualisation, Methodology, Formal analysis, Writing Original Draft.

RL: Conceptualisation, Writing- Review and Editing, Supervision.

## Funding information

This work was supported by the 10.13039/501100000269Economic and Social Research Council (grant ES/P010229/1 to RL); and the Economic and Social Research Council-10.13039/501100000268Biotechnology and Biological Sciences Research Council Soc-B Centre for Doctoral Training (grant ES/P000347/1 to NM).

## Declaration of competing interest

None.

## Data Availability

The authors do not have permission to share data.
