# Supplementary material for: Childhood adversity and cardiometabolic biomarkers in mid-adulthood in the 1958 British birth cohort
Source: SSM Popul Health. 2022 Oct 4;19:101260. doi: 10.1016/j.ssmph.2022.101260 (PMC9550530; doi:10.1016/j.ssmph.2022.101260)
Supplement: Multimedia component 1 [file mmc1.docx]

**Supplementary Table 1.** Regression coefficients (mean difference, % difference or OR) showing associations between adverse childhood experiences and cardiometabolic markers at age 44/45 in the NCDS (complete case analysis)

|  | Prospective childhood adversity | | Retrospective childhood adversity | |
| --- | --- | --- | --- | --- |
|  | *B* (95% CI)^a^ | *p* | *B* (95% CI)^a^ | *p* |
| Parental separation/divorce |  |  |  |  |
| HbA1c** | 0.47 (-1.16, 2.11) | 0.569 | 0.03 (-1.34, 1.41) | 0.963 |
| LDL cholesterol | **0.16 (0.01, 0.32)** | **0.041** | -0.01 (-0.14, 0.11) | 0.821 |
| HDL cholesterol | -0.04 (-0.10, 0.02) | 0.195 | -0.02 (-0.07, 0.03) | 0.481 |
| Total cholesterol | 0.13 (-0.05, 0.31) | 0.164 | -0.10 (-0.26, 0.05) | 0.184 |
| Triglycerides** | **5.82 (0.00, 11.63)** | **0.050** | 0.33 (-4.56, 5.22) | 0.895 |
| Systolic blood pressure | **2.57 (0.25, 4.88)** | **0.030** | 0.09 (-1.83, 2.01) | 0.927 |
| Diastolic blood pressure | **1.73 (0.13, 3.33)** | **0.034** | 0.41 (-0.93, 1.74) | 0.549 |
| BMI | 0.61 (-0.15, 1.37) | 0.117 | -0.13 (-0.77, 0.51) | 0.686 |
| Waist circumference | 1.32 (-0.38, 3.03) | 0.128 | 0.00 (-1.44, 1.43) | 0.996 |
| Metabolic syndrome*** | 1.11 (0.69, 1.78) | 0.660 | 0.94 (0.61, 1.44) | 0.770 |
| Parental substance misuse |  |  |  |  |
| HbA1c** | 2.65 (-1.62, 6.92) | 0.224 | -0.37 (-1.31, 0.57) | 0.438 |
| LDL cholesterol | **0.65 (0.20, 1.09)** | **0.004*** | 0.01 (-0.08, 0.10) | 0.813 |
| HDL cholesterol | 0.06 (-0.10, 0.22) | 0.469 | -0.03 (-0.06, 0.01) | 0.132 |
| Total cholesterol | **0.81 (0.32, 1.31)** | **0.001*** | -0.02 (-0.12, 0.09) | 0.738 |
| Triglycerides** | 13.41 (-2.55, 29.38) | 0.100 | 1.77 (-1.56, 5.10) | 0.297 |
| Systolic blood pressure | 0.45 (-6.07, 6.97) | 0.892 | -0.77 (-2.08, 0.55) | 0.252 |
| Diastolic blood pressure | 0.05 (-4.48, 4.59) | 0.981 | -0.20 (-1.10, 0.71) | 0.672 |
| BMI | -0.91 (-3.05, 1.23) | 0.404 | 0.19 (-0.24, 0.62) | 0.385 |
| Waist circumference | -0.39 (-5.27, 4.50) | 0.877 | 0.42 (-0.56, 1.39) | 0.400 |
| Metabolic syndrome*** | 1.54 (0.51, 4.69) | 0.447 | 1.21 (0.92, 1.58) | 0.169 |
| Parental mental illness |  |  |  |  |
| HbA1c** | 1.28 (-0.20, 2.75) | 0.091 | -0.34 (-1.04, 0.37) | 0.352 |
| LDL cholesterol | 0.02 (-0.12, 0.16) | 0.798 | 0.02 (-0.04, 0.09) | 0.491 |
| HDL cholesterol | 0.03 (-0.02, 0.08) | 0.272 | -0.01 (-0.03, 0.02) | 0.647 |
| Total cholesterol | 0.03 (-0.13, 0.19) | 0.753 | 0.00 (-0.07, 0.08) | 0.910 |
| Triglycerides** | -1.57 (-6.76, 3.63) | 0.554 | 0.42 (-2.08, 2.92) | 0.740 |
| Systolic blood pressure | -0.40 (-2.46, 1.66) | 0.705 | -0.43 (-1.42, 0.56) | 0.396 |
| Diastolic blood pressure | 0.34 (-1.09, 1.76) | 0.643 | -0.10 (-0.79, 0.59) | 0.773 |
| BMI | -0.06 (-0.74, 0.62) | 0.866 | -0.15 (-0.48, 0.17) | 0.360 |
| Waist circumference | -0.95 (-2.48, 0.57) | 0.221 | -0.16 (-0.89, 0.58) | 0.677 |
| Metabolic syndrome*** | 0.91 (0.58, 1.42) | 0.674 | 0.96 (0.78, 1.20) | 0.742 |
| Family conflict |  |  |  |  |
| HbA1c** | -0.13 (-1.86, 1.61) | 0.885 | 0.74 (-0.23, 1.72) | 0.135 |
| LDL cholesterol | **0.17 (0.01, 0.33)** | **0.042** | 0.01 (-0.08, 0.10) | 0.857 |
| HDL cholesterol | -0.02 (-0.09, 0.04) | 0.482 | **-0.04 (-0.08, -0.01)** | **0.022** |
| Total cholesterol | 0.10 (-0.09, 0.29) | 0.300 | -0.01 (-0.11, 0.10) | 0.891 |
| Triglycerides** | 1.67 (-4.48, 7.83) | 0.594 | 2.11 (-1.36, 5.58) | 0.234 |
| Systolic blood pressure | -1.08 (-3.51, 1.35) | 0.382 | **-1.56 (-2.92, -0.19)** | **0.025** |
| Diastolic blood pressure | 0.37 (-1.32, 2.06) | 0.671 | -0.76 (-1.71, 0.18) | 0.114 |
| BMI | -0.09 (-0.87, 0.68) | 0.812 | 0.14 (-0.30, 0.59) | 0.530 |
| Waist circumference | 0.17 (-1.65, 1.99) | 0.857 | 0.48 (-0.53, 1.49) | 0.352 |
| Metabolic syndrome*** | 0.69 (0.38, 1.25) | 0.219 | 1.23 (0.93, 1.63) | 0.139 |
| Witnessed abuse |  |  |  |  |
| HbA1c** | - |  | 0.40 (-1.08, 1.88) | 0.599 |
| LDL cholesterol | - |  | 0.08 (-0.06, 0.22) | 0.271 |
| HDL cholesterol | - |  | 0.00 (-0.06, 0.05) | 0.930 |
| Total cholesterol | - |  | 0.14 (-0.02, 0.30) | 0.097 |
| Triglycerides** | - |  | 3.50 (-1.77, 8.77) | 0.193 |
| Systolic blood pressure | - |  | 0.31 (-1.76, 2.38) | 0.770 |
| Diastolic blood pressure | - |  | 0.38 (-1.05, 1.82) | 0.601 |
| BMI | - |  | 0.29 (-0.39, 0.97) | 0.405 |
| Waist circumference | - |  | 0.78 (-0.75, 2.31) | 0.319 |
| Metabolic syndrome*** | - |  | 1.18 (0.78, 1.80) | 0.435 |
| Parental death |  |  |  |  |
| HbA1c** | 1.46 (-0.62, 3.54) | 0.169 | - |  |
| LDL cholesterol | 0.01 (-0.18, 0.21) | 0.899 | - |  |
| HDL cholesterol | -0.06 (-0.13, 0.02) | 0.158 | - |  |
| Total cholesterol | -0.15 (-0.39, 0.08) | 0.192 | - |  |
| Triglycerides** | -4.68 (-12.17, 2.80) | 0.220 | - |  |
| Systolic blood pressure | 1.47 (-1.38, 4.31) | 0.311 | - |  |
| Diastolic blood pressure | 1.08 (-0.89, 3.05) | 0.282 | - |  |
| BMI | 0.83 (-0.10, 1.75) | 0.081 | - |  |
| Waist circumference | 2.09 (-0.01, 4.19) | 0.051 | - |  |
| Metabolic syndrome*** | 1.58 (0.91, 2.73) | 0.101 | - |  |
| Parental offending |  |  |  |  |
| HbA1c** | **2.73 (1.21, 4.25)** | **<0.001*** | - |  |
| LDL cholesterol | 0.03 (-0.12, 0.18) | 0.714 | - |  |
| HDL cholesterol | -0.02 (-0.08, 0.03) | 0.396 | - |  |
| Total cholesterol | 0.01 (-0.15, 0.18) | 0.868 | - |  |
| Triglycerides** | **6.80 (1.35, 12.24)** | **0.014** | - |  |
| Systolic blood pressure | -0.61 (-2.76, 1.53) | 0.575 | - |  |
| Diastolic blood pressure | 0.43 (-1.06, 1.92) | 0.571 | - |  |
| BMI | 0.00 (-0.69, 0.69) | 0.999 | - |  |
| Waist circumference | 0.12 (-1.48, 1.72) | 0.883 | - |  |
| Metabolic syndrome*** | 1.15 (0.76, 1.74) | 0.508 | - |  |
| Physical neglect |  |  |  |  |
| HbA1c** | 0.75 (-0.85, 2.34) | 0.359 | - |  |
| LDL cholesterol | 0.13 (-0.01, 0.28) | 0.077 | - |  |
| HDL cholesterol | 0.01 (-0.05, 0.07) | 0.782 | - |  |
| Total cholesterol | 0.17 (0.00, 0.35) | 0.051 | - |  |
| Triglycerides** | **6.79 (1.14, 12.44)** | **0.019** | - |  |
| Systolic blood pressure | -0.37 (-2.60, 1.85) | 0.742 | - |  |
| Diastolic blood pressure | 0.35 (-1.19, 1.90) | 0.655 | - |  |
| BMI | -0.62 (-1.36, 0.13) | 0.107 | - |  |
| Waist circumference | -1.29 (-2.95, 0.36) | 0.126 | - |  |
| Metabolic syndrome*** | 1.03 (0.66, 1.60) | 0.909 | - |  |
| Emotional neglect |  |  |  |  |
| HbA1c** | - |  | 0.87 (-0.15, 1.89) | 0.094 |
| LDL cholesterol | - |  | 0.01 (-0.09, 0.10) | 0.857 |
| HDL cholesterol | - |  | **-0.05 (-0.09, -0.02)** | **0.006** |
| Total cholesterol | - |  | 0.00 (-0.11, 0.11) | 0.964 |
| Triglycerides** | - |  | 2.90 (-0.73, 6.53) | 0.117 |
| Systolic blood pressure | - |  | -0.81 (-2.24, 0.62) | 0.267 |
| Diastolic blood pressure | - |  | -0.45 (-1.45, 0.54) | 0.369 |
| BMI | - |  | 0.11 (-0.36, 0.57) | 0.653 |
| Waist circumference | - |  | -0.13 (-1.19, 0.94) | 0.813 |
| Metabolic syndrome*** | - |  | 1.22 (0.91, 1.63) | 0.177 |
| Physical abuse |  |  |  |  |
| HbA1c** | - |  | **1.60 (0.15, 3.05)** | **0.031** |
| LDL cholesterol | - |  | **0.14 (0.00, 0.28)** | **0.048** |
| HDL cholesterol | - |  | -0.03 (-0.08, 0.02) | 0.279 |
| Total cholesterol | - |  | 0.13 (-0.03, 0.29) | 0.112 |
| Triglycerides** | - |  | 2.29 (-2.86, 7.43) | 0.384 |
| Systolic blood pressure | - |  | -0.32 (-2.33, 1.69) | 0.753 |
| Diastolic blood pressure | - |  | -0.10 (-1.49, 1.30) | 0.893 |
| BMI | - |  | 0.60 (-0.05, 1.26) | 0.072 |
| Waist circumference | - |  | 1.27 (-0.22, 2.75) | 0.095 |
| Metabolic syndrome*** | - |  | 1.10 (0.73, 1.67) | 0.641 |
| Sexual abuse |  |  |  |  |
| HbA1c** | - |  | 1.17 (-2.50, 4.83) | 0.533 |
| LDL cholesterol | - |  | 0.20 (-0.15, 0.55) | 0.264 |
| HDL cholesterol | - |  | -0.07 (-0.20, 0.07) | 0.351 |
| Total cholesterol | - |  | 0.14 (-0.28, 0.56) | 0.515 |
| Triglycerides** | - |  | 4.46 (-9.11, 18.03) | 0.519 |
| Systolic blood pressure | - |  | -0.60 (-5.71, 4.52) | 0.819 |
| Diastolic blood pressure | - |  | 0.39 (-3.15, 3.94) | 0.829 |
| BMI | - |  | -0.18 (-1.88, 1.53) | 0.838 |
| Waist circumference | - |  | 0.33 (-3.43, 4.09) | 0.863 |
| Metabolic syndrome*** | - |  | 0.55 (0.13, 2.30) | 0.428 |
| Psychological abuse |  |  |  |  |
| HbA1c** | - |  | 0.90 (-0.23, 2.02) | 0.118 |
| LDL cholesterol | - |  | 0.02 (-0.08, 0.13) | 0.660 |
| HDL cholesterol | - |  | **-0.05 (-0.09, -0.01)** | **0.025** |
| Total cholesterol | - |  | -0.01 (-0.13, 0.12) | 0.923 |
| Triglycerides** | - |  | 1.99 (-2.01, 6.00) | 0.329 |
| Systolic blood pressure | - |  | -0.25 (-1.81, 1.32) | 0.757 |
| Diastolic blood pressure | - |  | 0.01 (-1.07, 1.10) | 0.984 |
| BMI | - |  | 0.18 (-0.33, 0.70) | 0.491 |
| Waist circumference | - |  | 0.83 (-0.33, 1.99) | 0.160 |
| Metabolic syndrome*** | - |  | 1.10 (0.79, 1.53) | 0.559 |

*Note.* Associations with *p​≤*0.05 are presented in bold-face.

*Findings significant under Bonferroni correction (*p​≤*0.005).

**Results presented as % differences (95% CI) as outcomes were positively skewed and log transformed prior to analysis.

***Results presented as ORs (95% CI).

^a^ Models are adjusted for sex, father’s occupation at birth, maternal education level at birth, household overcrowding at 7 years, birth weight, gestational age, maternal age at birth, maternal smoking during pregnancy and mother’s BMI before childbirth.

ACE, adverse childhood experience; BMI, body mass index; LDL, low-density lipoprotein; HDL, high-density lipoprotein; OR, odds ratio.

**Supplementary Table 2.** Regression coefficients (mean difference, % difference or OR) showing associations between ACE scores and cardiometabolic markers at age 44/45 in the NCDS (complete case analysis)

|  | Prospective childhood adversity | | | Retrospective childhood adversity | | |
| --- | --- | --- | --- | --- | --- | --- |
|  |  | *B* (95% CI)^a^ | *p* |  | *B* (95% CI)^a^ | *p* |
| HbA1c** |  |  |  |  |  |  |
|  | 0 ACEs | Ref |  | 0 ACEs | Ref |  |
|  | 1 ACEs | 0.05 (-1.01, 1.11) | 0.924 | 1 ACEs | -0.08 (-0.99, 0.83) | 0.869 |
|  | 2+ ACEs | **2.12 (0.35, 3.89)** | **0.019** | 2 ACEs | -0.54 (-1.69, 0.61) | 0.354 |
|  |  |  |  | 3 ACEs | -0.86 (-2.55, 0.83) | 0.509 |
|  |  |  |  | 4+ ACEs | 0.47 (-0.93, 1.86) | 0.509 |
| LDL cholesterol |  |  |  |  |  |  |
|  | 0 ACEs | Ref |  | 0 ACEs | Ref |  |
|  | 1 ACEs | **0.15 (0.05, 0.25)** | **0.003*** | 1 ACEs | -0.02 (-0.11, 0.06) | 0.628 |
|  | 2+ ACEs | **0.21 (0.04, 0.38)** | **0.015** | 2 ACEs | -0.04 (-0.15, 0.07) | 0.515 |
|  |  |  |  | 3 ACEs | -0.14 (-0.30, 0.02) | 0.092 |
|  |  |  |  | 4+ ACEs | 0.05 (-0.08, 0.18) | 0.452 |
| HDL cholesterol |  |  |  |  |  |  |
|  | 0 ACEs | Ref |  | 0 ACEs | Ref |  |
|  | 1 ACEs | 0.01 (-0.03, 0.05) | 0.569 | 1 ACEs | **-0.04 (-0.07, -0.01)** | **0.024** |
|  | 2+ ACEs | 0.00 (-0.07, 0.06) | 0.953 | 2 ACEs | -0.01 (-0.05, 0.03) | 0.680 |
|  |  |  |  | 3 ACEs | -0.06 (-0.12, 0.00) | 0.070 |
|  |  |  |  | 4+ ACEs | -0.04 (-0.09, 0.01) | 0.121 |
| Total cholesterol |  |  |  |  |  |  |
|  | 0 ACEs | Ref |  | 0 ACEs | Ref |  |
|  | 1 ACEs | **0.13 (0.01, 0.24)** | **0.035** | 1 ACEs | -0.06 (-0.16, 0.04) | 0.222 |
|  | 2+ ACEs | 0.19 (0.00, 0.39) | 0.055 | 2 ACEs | -0.07 (-0.20, 0.06) | 0.306 |
|  |  |  |  | 3 ACEs | **-0.20 (-0.39, -0.01)** | **0.035** |
|  |  |  |  | 4+ ACEs | 0.01 (-0.15, 0.16) | 0.905 |
| Triglycerides** |  |  |  |  |  |  |
|  | 0 ACEs | Ref |  | 0 ACEs | Ref |  |
|  | 1 ACEs | -0.21 (-3.97, 3.55) | 0.914 | 1 ACEs | 1.53 (-1.71, 4.76) | 0.355 |
|  | 2+ ACEs | **6.34 (0.00, 12.68)** | **0.050** | 2 ACEs | 0.78 (-3.35, 4.92) | 0.711 |
|  |  |  |  | 3 ACEs | -1.27 (-7.33, 4.78) | 0.680 |
|  |  |  |  | 4+ ACEs | 2.19 (-2.78, 7.16) | 0.388 |
| Systolic blood pressure |  |  |  |  |  |  |
|  | 0 ACEs | Ref |  | 0 ACEs | Ref |  |
|  | 1 ACEs | -0.75 (-2.25, 0.74) | 0.322 | 1 ACEs | 0.49 (-0.79, 1.77) | 0.451 |
|  | 2+ ACEs | 1.32 (-1.15, 3.78) | 0.295 | 2 ACEs | -1.25 (-2.87, 0.38) | 0.133 |
|  |  |  |  | 3 ACEs | -1.88 (-4.26, 0.49) | 0.121 |
|  |  |  |  | 4+ ACEs | -1.69 (-3.66, 0.29) | 0.095 |
| Diastolic blood pressure |  |  |  |  |  |  |
|  | 0 ACEs | Ref |  | 0 ACEs | Ref |  |
|  | 1 ACEs | -0.36 (-1.40, 0.68) | 0.500 | 1 ACEs | 0.57 (-0.32, 1.45) | 0.213 |
|  | 2+ ACEs | 1.53 (-0.19, 3.24) | 0.081 | 2 ACEs | -0.21 (-1.34, 0.91) | 0.711 |
|  |  |  |  | 3 ACEs | -1.14 (-2.79, 0.50) | 0.174 |
|  |  |  |  | 4+ ACEs | -0.93 (-2.30, 0.44) | 0.184 |
| BMI |  |  |  |  |  |  |
|  | 0 ACEs | Ref |  | 0 ACEs | Ref |  |
|  | 1 ACEs | -0.22 (-0.72, 0.27) | 0.377 | 1 ACEs | -0.11 (-0.53, 0.31) | 0.608 |
|  | 2+ ACEs | 0.20 (-0.59, 0.98) | 0.618 | 2 ACEs | 0.06 (-0.48, 0.60) | 0.819 |
|  |  |  |  | 3 ACEs | -0.15 (-0.91, 0.61) | 0.698 |
|  |  |  |  | 4+ ACEs | -0.23 (-0.88, 0.42) | 0.494 |
| Waist circumference |  |  |  |  |  |  |
|  | 0 ACEs | Ref |  | 0 ACEs | Ref |  |
|  | 1 ACEs | -0.49 (-1.60, 0.62) | 0.384 | 1 ACEs | 0.13 (-0.83, 1.09) | 0.785 |
|  | 2+ ACEs | -0.25 (-2.08, 1.58) | 0.789 | 2 ACEs | 0.68 (-0.53, 1.89) | 0.269 |
|  |  |  |  | 3 ACEs | -0.26 (-2.04, 1.51) | 0.770 |
|  |  |  |  | 4+ ACEs | -0.68 (-2.15, 0.80) | 0.368 |
| Metabolic syndrome*** |  |  |  |  |  |  |
|  | 0 ACEs | Ref |  | 0 ACEs | Ref |  |
|  | 1 ACEs | 0.88 (0.64, 1.22) | 0.449 | 1 ACEs | 1.10 (0.83, 1.44) | 0.510 |
|  | 2+ ACEs | 1.32 (0.82, 2.13) | 0.253 | 2 ACEs | 0.96 (0.66, 1.39) | 0.830 |
|  |  |  |  | 3 ACEs | 1.19 (0.72, 1.95) | 0.499 |
|  |  |  |  | 4+ ACEs | 1.20 (0.80, 1.80) | 0.386 |

*Note.* Associations with *p​≤*0.05 are presented in bold-face.

*Findings significant under Bonferroni correction (*p​≤*0.005).

**Results presented as % differences (95% CI) as outcomes were positively skewed and log transformed prior to analysis.

***Results presented as ORs (95% CI).

^a^ Models are adjusted for sex, father’s occupation at birth, maternal education level at birth, household overcrowding at 7 years, birth weight, gestational age, maternal age at birth, maternal smoking during pregnancy and mother’s BMI before childbirth.

ACE, adverse childhood experience; BMI, body mass index; LDL, low-density lipoprotein; HDL, high-density lipoprotein; OR, odds ratio.

**Supplementary Table 3.** Regression coefficients (mean difference, % difference or OR) showing associations between ACEs and cardiometabolic markers at age 44/45 in the NCDS (excluding, not correcting, for those on treatment)

|  | Prospective childhood adversity | | Retrospective childhood adversity | |
| --- | --- | --- | --- | --- |
|  | *B* (95% CI)^a^ | *p* | *B* (95% CI)^a^ | *p* |
| Parental separation/divorce |  |  |  |  |
| HbA1c**, *N* = 8359 | 0.58 (-0.30, 1.46) | 0.195 | 0.23 (-0.59, 1.04) | 0.585 |
| LDL cholesterol, *N* = 8383 | 0.01 (-0.09, 0.10) | 0.893 | -0.04 (-0.13, 0.04) | 0.300 |
| HDL cholesterol, *N* = 8383 | **-0.07 (-0.11, -0.03)** | **0.001*** | **-0.05 (-0.09, -0.02)** | **0.002*** |
| Total cholesterol, *N* = 8383 | 0.00 (-0.11, 0.11) | 0.992 | -0.08 (-0.18, 0.01) | 0.094 |
| Triglycerides**, *N* = 8383 | 3.50 (-0.50, 7.49) | 0.086 | 1.16 (-2.14, 4.47) | 0.488 |
| Systolic blood pressure, *N* = 8120 | -0.14 (-1.64, 1.37) | 0.858 | -0.85 (-2.27, 0.58) | 0.243 |
| Diastolic blood pressure, *N* = 8120 | 0.20 (-0.85, 1.24) | 0.711 | -0.30 (-1.25, 0.66) | 0.545 |
| Metabolic syndrome***, *N* = 7942 | 1.19 (0.88, 1.60) | 0.267 | 1.05 (0.79, 1.38) | 0.751 |
| Parental substance misuse |  |  |  |  |
| HbA1c**, *N* = 8359 | 0.46 (-1.89, 2.82) | 0.696 | -0.26 (-0.85, 0.32) | 0.375 |
| LDL cholesterol, *N* = 8383 | 0.00 (-0.24, 0.24) | 0.994 | -0.02 (-0.09, 0.05) | 0.528 |
| HDL cholesterol, *N* = 8383 | -0.04 (-0.15, 0.08) | 0.545 | -0.01 (-0.04, 0.02) | 0.514 |
| Total cholesterol, *N* = 8383 | 0.07 (-0.24, 0.38) | 0.658 | -0.02 (-0.10, 0.05) | 0.519 |
| Triglycerides**, *N* = 8383 | 4.95 (-5.29, 15.20) | 0.339 | 0.59 (-1.84, 3.02) | 0.634 |
| Systolic blood pressure, *N* = 8120 | 0.10 (-5.07, 5.27) | 0.970 | -0.36 (-1.35, 0.64) | 0.480 |
| Diastolic blood pressure, *N* = 8120 | -0.05 (-3.15, 3.06) | 0.976 | -0.14 (-0.83, 0.55) | 0.689 |
| Metabolic syndrome***, *N* = 7942 | 1.05 (0.45, 2.46) | 0.915 | 1.05 (0.85, 1.30) | 0.639 |
| Parental mental illness |  |  |  |  |
| HbA1c**, *N* = 8359 | -0.07 (-0.99, 0.85) | 0.874 | -0.12 (-0.56, 0.32) | 0.586 |
| LDL cholesterol, *N* = 8383 | 0.06 (-0.05, 0.17) | 0.259 | 0.01 (-0.04, 0.06) | 0.632 |
| HDL cholesterol, *N* = 8383 | 0.03 (-0.01, 0.07) | 0.172 | 0.01 (-0.01, 0.03) | 0.427 |
| Total cholesterol, *N* = 8383 | 0.07 (-0.05, 0.19) | 0.235 | 0.02 (-0.04, 0.07) | 0.568 |
| Triglycerides**, *N* = 8383 | -2.05 (-5.76, 1.66) | 0.278 | -0.47 (-2.35, 1.42) | 0.626 |
| Systolic blood pressure, *N* = 8120 | -0.84 (-2.47, 0.78) | 0.307 | 0.01 (-0.76, 0.78) | 0.985 |
| Diastolic blood pressure, *N* = 8120 | -0.02 (-1.15, 1.12) | 0.975 | 0.20 (-0.33, 0.73) | 0.462 |
| Metabolic syndrome***, *N* = 7942 | 0.95 (0.68, 1.32) | 0.743 | 0.99 (0.84, 1.17) | 0.928 |
| Family conflict |  |  |  |  |
| HbA1c**, *N* = 8359 | 0.21 (-0.92, 1.35) | 0.710 | 0.48 (-0.13, 1.08) | 0.121 |
| LDL cholesterol, *N* = 8383 | 0.05 (-0.06, 0.16) | 0.360 | 0.02 (-0.05, 0.09) | 0.522 |
| HDL cholesterol, *N* = 8383 | **-0.05 (-0.10, -0.001)** | **0.046** | **-0.03 (-0.06, -0.01)** | **0.018** |
| Total cholesterol, *N* = 8383 | 0.03 (-0.09, 0.16) | 0.594 | 0.00 (-0.08, 0.08) | 0.966 |
| Triglycerides**, *N* = 8383 | 1.91 (-2.51, 6.34) | 0.394 | 0.79 (-1.69, 3.28) | 0.532 |
| Systolic blood pressure, *N* = 8120 | -0.69 (-2.44, 1.05) | 0.435 | **-1.57 (-2.58, -0.55)** | **0.002*** |
| Diastolic blood pressure, *N* = 8120 | 0.08 (-1.12, 1.29) | 0.892 | -0.67 (-1.37, 0.04) | 0.063 |
| Metabolic syndrome***, *N* = 7942 | 1.04 (0.72, 1.48) | 0.848 | 1.78 (0.96, 1.45) | 0.123 |
| Witnessed abuse |  |  |  |  |
| HbA1c**, *N* = 8359 | - |  | -0.20 (-1.13, 0.74) | 0.675 |
| LDL cholesterol, *N* = 8383 | - |  | 0.08 (-0.02, 0.17) | 0.123 |
| HDL cholesterol, *N* = 8383 | - |  | -0.03 (-0.07, 0.01) | 0.181 |
| Total cholesterol, *N* = 8383 | - |  | 0.07 (-0.04, 0.18) | 0.215 |
| Triglycerides**, *N* = 8383 | - |  | 2.42 (-1.30, 6.13) | 0.202 |
| Systolic blood pressure, *N* = 8120 | - |  | -0.81 (-2.29, 0.68) | 0.287 |
| Diastolic blood pressure, *N* = 8120 | - |  | 0.09 (-0.93, 1.12) | 0.857 |
| Metabolic syndrome***, *N* = 7942 | - |  | 1.16 (0.85, 1.58) | 0.337 |
| Parental death |  |  |  |  |
| HbA1c**, *N* = 8359 | 0.21 (-0.85, 1.26) | 0.700 | - |  |
| LDL cholesterol, *N* = 8383 | 0.06 (-0.07, 0.18) | 0.390 | - |  |
| HDL cholesterol, *N* = 8383 | -0.04 (-0.10, 0.01) | 0.100 | - |  |
| Total cholesterol, *N* = 8383 | -0.03 (-0.17, 0.11) | 0.681 | - |  |
| Triglycerides**, *N* = 8383 | -1.96 (-6.93, 3.02) | 0.438 | - |  |
| Systolic blood pressure, *N* = 8120 | -0.50 (-2.43, 1.43) | 0.610 | - |  |
| Diastolic blood pressure, *N* = 8120 | -0.01 (-1.36, 1.34) | 0.987 | - |  |
| Metabolic syndrome***, *N* = 7942 | 1.30 (0.88, 1.92) | 0.181 | - |  |
| Parental offending |  |  |  |  |
| HbA1c**, *N* = 8359 | **1.26 (0.33, 2.19)** | **0.008** | - |  |
| LDL cholesterol, *N* = 8383 | -0.02 (-0.12, 0.07) | 0.629 | - |  |
| HDL cholesterol, *N* = 8383 | -0.02 (-0.06, 0.02) | 0.285 | - |  |
| Total cholesterol, *N* = 8383 | 0.06 (-0.06, 0.17) | 0.332 | - |  |
| Triglycerides**, *N* = 8383 | **5.62 (2.01, 9.22)** | **0.002*** | - |  |
| Systolic blood pressure, *N* = 8120 | 0.67 (-0.88, 2.22) | 0.399 | - |  |
| Diastolic blood pressure, *N* = 8120 | 0.60 (-0.45, 1.66) | 0.262 | - |  |
| Metabolic syndrome***, *N* = 7942 | **1.37 (1.02, 1.84)** | **0.037** | - |  |
| Physical neglect |  |  |  |  |
| HbA1c**, *N* = 8359 | 0.60 (-0.54, 1.75) | 0.295 | - |  |
| LDL cholesterol, *N* = 8383 | -0.01 (-0.12, 0.09) | 0.803 | - |  |
| HDL cholesterol, *N* = 8383 | **-0.07 (-0.11, -0.02)** | **0.002*** | - |  |
| Total cholesterol, *N* = 8383 | 0.04 (-0.08, 0.16) | 0.498 | - |  |
| Triglycerides**, *N* = 8383 | **7.13 (2.90, 11.36)** | **0.001*** | - |  |
| Systolic blood pressure, *N* = 8120 | -0.36 (-2.02, 1.30) | 0.673 | - |  |
| Diastolic blood pressure, *N* = 8120 | -0.25 (-1.38, 0.89) | 0.669 | - |  |
| Metabolic syndrome***, *N* = 7942 | 0.98 (0.71, 1.37) | 0.920 | - |  |
| Emotional neglect |  |  |  |  |
| HbA1c**, *N* = 8359 | - |  | 0.34 (-0.28, 0.96) | 0.283 |
| LDL cholesterol, *N* = 8383 | - |  | 0.01 (-0.06, 0.08) | 0.806 |
| HDL cholesterol, *N* = 8383 | - |  | **-0.05 (-0.08, -0.02)** | **<0.001*** |
| Total cholesterol, *N* = 8383 | - |  | -0.02 (-0.10, 0.05) | 0.554 |
| Triglycerides**, *N* = 8383 | - |  | 1.69 (-0.88, 4.26) | 0.197 |
| Systolic blood pressure, *N* = 8120 | - |  | **-1.47 (-2.53, -0.41)** | **0.007** |
| Diastolic blood pressure, *N* = 8120 | - |  | -0.43 (-1.16, 0.31) | 0.256 |
| Metabolic syndrome***, *N* = 7942 | - |  | 1.17 (0.94, 1.45) | 0.169 |
| Physical abuse |  |  |  |  |
| HbA1c**, *N* = 8359 | - |  | 0.96 (-0.04, 1.96) | 0.059 |
| LDL cholesterol, *N* = 8383 | - |  | 0.08 (-0.02, 0.18) | 0.098 |
| HDL cholesterol, *N* = 8383 | - |  | -0.03 (-0.07, 0.01) | 0.098 |
| Total cholesterol, *N* = 8383 | - |  | 0.06 (-0.05, 0.18) | 0.293 |
| Triglycerides**, *N* = 8383 | - |  | 1.49 (-2.15, 5.13) | 0.423 |
| Systolic blood pressure, *N* = 8120 | - |  | -0.52 (-1.98, 0.94) | 0.485 |
| Diastolic blood pressure, *N* = 8120 | - |  | -0.20 (-1.21, 0.81) | 0.696 |
| Metabolic syndrome***, *N* = 7942 | - |  | 1.19 (0.89, 1.60) | 0.247 |
| Sexual abuse |  |  |  |  |
| HbA1c**, *N* = 8359 | - |  | 0.47 (-1.25, 2.20) | 0.590 |
| LDL cholesterol, *N* = 8383 | - |  | 0.11 (-0.07, 0.30) | 0.236 |
| HDL cholesterol, *N* = 8383 | - |  | -0.02 (-0.10, 0.06) | 0.592 |
| Total cholesterol, *N* = 8383 | - |  | 0.03 (-0.18, 0.25) | 0.767 |
| Triglycerides**, *N* = 8383 | - |  | -3.46 (-10.83, 3.91) | 0.357 |
| Systolic blood pressure, *N* = 8120 | - |  | **-3.02 (-6.04, 0.00)** | **0.050** |
| Diastolic blood pressure, *N* = 8120 | - |  | -1.99 (-4.08, 0.10) | 0.062 |
| Metabolic syndrome***, *N* = 7942 | - |  | 0.90 (0.46, 1.79) | 0.772 |
| Psychological abuse |  |  |  |  |
| HbA1c**, *N* = 8359 | - |  | 0.50 (-0.25, 1.25) | 0.188 |
| LDL cholesterol, *N* = 8383 | - |  | 0.06 (-0.01, 0.14) | 0.108 |
| HDL cholesterol, *N* = 8383 | - |  | **-0.05 (-0.08, -0.02)** | **0.002*** |
| Total cholesterol, *N* = 8383 | - |  | 0.02 (-0.07, 0.11) | 0.680 |
| Triglycerides**, *N* = 8383 | - |  | 1.04 (-1.87, 3.94) | 0.484 |
| Systolic blood pressure, *N* = 8120 | - |  | -0.35 (-1.52, 0.82) | 0.562 |
| Diastolic blood pressure, *N* = 8120 | - |  | 0.19 (-0.62, 1.00) | 0.642 |
| Metabolic syndrome***, *N* = 7942 | - |  | 1.15 (0.90, 1.45) | 0.261 |

*Note.* Associations with *p​≤*0.05 are presented in bold-face.

*Findings significant under Bonferroni correction (*p​≤*0.005).

**Results presented as % differences (95% CI) as outcomes were positively skewed and log transformed prior to analysis.

***Results presented as ORs (95% CI).

^a^ Models are adjusted for sex, father’s occupation at birth, maternal education level at birth, household overcrowding at 7 years, birth weight, gestational age, maternal age at birth, maternal smoking during pregnancy and mother’s BMI before childbirth.

ACE, adverse childhood experience; BMI, body mass index; LDL, low-density lipoprotein; HDL, high-density lipoprotein; OR, odds ratio.

**Supplementary Table 4.** Regression coefficients (mean difference, % difference or OR) showing associations between ACE scores and cardiometabolic markers at age 44/45 in the NCDS (excluding, not correcting, for those on treatment)

|  | Prospective childhood adversity | | | Retrospective childhood adversity | | |
| --- | --- | --- | --- | --- | --- | --- |
|  |  | *B* (95% CI)^a^ | *p* |  | *B* (95% CI)^a^ | *p* |
| HbA1c**, *N* = 8359 |  |  |  |  |  |  |
|  | 0 ACEs | Ref |  | 0 ACEs | Ref |  |
|  | 1 ACEs | 0.44 (-0.15, 1.04) | 0.142 | 1 ACEs | -0.04 (-0.57, 0.48) | 0.871 |
|  | 2+ ACEs | 0.60 (-0.34, 1.55) | 0.208 | 2 ACEs | -0.23 (-0.89, 0.42) | 0.481 |
|  |  |  |  | 3 ACEs | -0.36 (-1.31, 0.59) | 0.456 |
|  |  |  |  | 4+ ACEs | 0.64 (-0.15, 1.43) | 0.109 |
| LDL cholesterol, *N* = 8383 |  |  |  |  |  |  |
|  | 0 ACEs | Ref |  | 0 ACEs | Ref |  |
|  | 1 ACEs | **0.07 (0.00, 0.14)** | **0.035** | 1 ACEs | 0.01 (-0.05, 0.07) | 0.644 |
|  | 2+ ACEs | -0.01 (-0.10, 0.08) | 0.836 | 2 ACEs | 0.00 (-0.08, 0.07) | 0.975 |
|  |  |  |  | 3 ACEs | -0.07 (-0.17, 0.04) | 0.215 |
|  |  |  |  | 4+ ACEs | 0.04 (-0.04, 0.12) | 0.333 |
| HDL cholesterol, *N* = 8383 |  |  |  |  |  |  |
|  | 0 ACEs | Ref |  | 0 ACEs | Ref |  |
|  | 1 ACEs | -0.02 (-0.05, 0.00) | 0.092 | 1 ACEs | -0.01 (-0.04, 0.01) | 0.277 |
|  | 2+ ACEs | **-0.06 (-0.10, -0.02)** | **0.002*** | 2 ACEs | 0.00 (-0.03, 0.03) | 0.952 |
|  |  |  |  | 3 ACEs | -0.02 (-0.07, 0.02) | 0.287 |
|  |  |  |  | 4+ ACEs | **-0.04 (-0.08, -0.01)** | **0.011** |
| Total cholesterol, *N* = 8383 |  |  |  |  |  |  |
|  | 0 ACEs | Ref |  | 0 ACEs | Ref |  |
|  | 1 ACEs | **0.08 (0.01, 0.16)** | **0.020** | 1 ACEs | 0.03 (-0.04, 0.09) | 0.465 |
|  | 2+ ACEs | 0.01 (-0.10, 0.11) | 0.877 | 2 ACEs | 0.01 (-0.08, 0.09) | 0.901 |
|  |  |  |  | 3 ACEs | -0.11 (-0.22, 0.01) | 0.080 |
|  |  |  |  | 4+ ACEs | 0.01 (-0.09, 0.10) | 0.906 |
| Triglycerides**, *N* = 8383 |  |  |  |  |  |  |
|  | 0 ACEs | Ref |  | 0 ACEs | Ref |  |
|  | 1 ACEs | 2.15 (-0.25, 4.55) | 0.080 | 1 ACEs | 1.38 (-0.95, 3.70) | 0.246 |
|  | 2+ ACEs | **4.14 (0.27, 8.02)** | **0.036** | 2 ACEs | 0.74 (-2.07, 3.55) | 0.606 |
|  |  |  |  | 3 ACEs | -1.36 (-5.30, 2.57) | 0.497 |
|  |  |  |  | 4+ ACEs | 1.11 (-1.93, 4.16) | 0.472 |
| Systolic blood pressure, *N* = 8120 |  |  |  |  |  |  |
|  | 0 ACEs | Ref |  | 0 ACEs | Ref |  |
|  | 1 ACEs | -0.08 (-1.12, 0.95) | 0.875 | 1 ACEs | 0.04 (-0.89, 0.98) | 0.927 |
|  | 2+ ACEs | -0.15 (-1.67, 1.38) | 0.849 | 2 ACEs | -0.34 (-1.50, 0.81) | 0.562 |
|  |  |  |  | 3 ACEs | **-2.14 (-3.76, -0.52)** | **0.010** |
|  |  |  |  | 4+ ACEs | -0.69 (-1.97, 0.59) | 0.289 |
| Diastolic blood pressure, *N* = 8120 |  |  |  |  |  |  |
|  | 0 ACEs | Ref |  | 0 ACEs | Ref |  |
|  | 1 ACEs | 0.08 (-0.64, 0.79) | 0.838 | 1 ACEs | 0.19 (-0.46, 0.84) | 0.568 |
|  | 2+ ACEs | 0.30 (-0.74, 1.35) | 0.567 | 2 ACEs | 0.15 (-0.65, 0.94) | 0.712 |
|  |  |  |  | 3 ACEs | **-1.20 (-2.32, -0.07)** | **0.037** |
|  |  |  |  | 4+ ACEs | -0.03 (-0.91, 0.85) | 0.944 |
| Metabolic syndrome***, *N* = 7942 |  |  |  |  |  |  |
|  | 0 ACEs | Ref |  | 0 ACEs | Ref |  |
|  | 1 ACEs | 0.99 (0.80, 1.23) | 0.938 | 1 ACEs | 1.13 (0.93, 1.37) | 0.229 |
|  | 2+ ACEs | 1.29 (0.97, 1.73) | 0.085 | 2 ACEs | 1.00 (0.77, 1.29) | 0.983 |
|  |  |  |  | 3 ACEs | 1.03 (0.73, 1.44) | 0.875 |
|  |  |  |  | 4+ ACEs | 1.22 (0.93, 1.59) | 0.146 |

*Note.* Associations with *p​≤*0.05 are presented in bold-face.

*Findings significant under Bonferroni correction (*p​≤*0.005).

**Results presented as % differences (95% CI) as outcomes were positively skewed and log transformed prior to analysis.

***Results presented as ORs (95% CI).

^a^ Models are adjusted for sex, father’s occupation at birth, maternal education level at birth, household overcrowding at 7 years, birth weight, gestational age, maternal age at birth, maternal smoking during pregnancy and mother’s BMI before childbirth.

ACE, adverse childhood experience; BMI, body mass index; LDL, low-density lipoprotein; HDL, high-density lipoprotein; OR, odds ratio.

**Supplementary Table 5.** Regression coefficients (mean difference, % difference or OR) showing associations between ACEs and cardiometabolic markers at age 44/45 in the NCDS (adjusting, not correcting, for those on treatment)

|  | Prospective childhood adversity | | Retrospective childhood adversity | |
| --- | --- | --- | --- | --- |
|  | *B* (95% CI)^a^ | *p* | *B* (95% CI)^a^ | *p* |
| Parental separation/divorce |  |  |  |  |
| HbA1c** | 0.68 (-0.23, 1.59) | 0.142 | 0.10 (-0.75, 0.95) | 0.821 |
| LDL cholesterol | 0.01 (-0.08, 0.10) | 0.792 | -0.04 (-0.12, 0.05) | 0.416 |
| HDL cholesterol | **-0.06 (-0.10, -0.02)** | **0.002*** | **-0.05 (-0.08, -0.02)** | **0.004*** |
| Total cholesterol | 0.01 (-0.10, 0.12) | 0.845 | -0.07 (-0.16, 0.03) | 0.174 |
| Triglycerides** | 3.42 (-0.58, 7.43) | 0.094 | 1.18 (-2.13, 4.48) | 0.484 |
| Systolic blood pressure | 0.19 (-1.29, 1.67) | 0.799 | -0.54 (-1.98, 0.90) | 0.461 |
| Diastolic blood pressure | 0.26 (-0.76, 1.29) | 0.613 | -0.18 (-1.14, 0.78) | 0.714 |
| Metabolic syndrome*** | 1.14 (0.86, 1.52) | 0.355 | 1.00 (0.77, 1.31) | 0.982 |
| Parental substance misuse |  |  |  |  |
| HbA1c** | 0.67 (-1.87, 3.21) | 0.602 | -0.48 (-1.09, 0.14) | 0.131 |
| LDL cholesterol | 0.05 (-0.21, 0.30) | 0.702 | -0.03 (-0.09, 0.04) | 0.442 |
| HDL cholesterol | 0.00 (-0.12, 0.11) | 0.932 | -0.01 (-0.04, 0.02) | 0.484 |
| Total cholesterol | 0.16 (-0.16, 0.48) | 0.323 | -0.03 (-0.11, 0.04) | 0.423 |
| Triglycerides** | 5.50 (-4.88, 15.88) | 0.295 | 0.52 (-1.91, 2.96) | 0.673 |
| Systolic blood pressure | 0.25 (-4.53, 5.03) | 0.917 | -0.39 (-1.37, 0.58) | 0.428 |
| Diastolic blood pressure | -0.11 (-3.00, 2.77) | 0.939 | -0.15 (-0.82, 0.52) | 0.662 |
| Metabolic syndrome*** | 1.13 (0.52, 2.43) | 0.761 | 1.04 (0.85, 1.27) | 0.678 |
| Parental mental illness |  |  |  |  |
| HbA1c** | 0.06 (-0.91, 1.03) | 0.900 | -0.25 (-0.71, 0.21) | 0.288 |
| LDL cholesterol | 0.05 (-0.05, 0.16) | 0.308 | 0.01 (-0.04, 0.06) | 0.691 |
| HDL cholesterol | 0.03 (-0.01, 0.07) | 0.174 | 0.01 (-0.01, 0.03) | 0.390 |
| Total cholesterol | 0.06 (-0.06, 0.19) | 0.294 | 0.01 (-0.04, 0.07) | 0.626 |
| Triglycerides** | -2.05 (-5.76, 1.67) | 0.280 | -0.52 (-2.41, 1.37) | 0.592 |
| Systolic blood pressure | -0.67 (-2.23, 0.90) | 0.404 | -0.08 (-0.84, 0.67) | 0.829 |
| Diastolic blood pressure | -0.05 (-1.14, 1.05) | 0.936 | 0.16 (-0.36, 0.68) | 0.539 |
| Metabolic syndrome*** | 0.91 (0.66, 1.25) | 0.550 | 0.98 (0.84, 1.14) | 0.797 |
| Family conflict |  |  |  |  |
| HbA1c** | 0.54 (-0.67, 1.75) | 0.373 | 0.37 (-0.26, 0.99) | 0.246 |
| LDL cholesterol | 0.06 (-0.06, 0.18) | 0.317 | 0.03 (-0.04, 0.10) | 0.417 |
| HDL cholesterol | -0.04 (-0.10, 0.01) | 0.078 | **-0.03 (-0.06, -0.002)** | **0.037** |
| Total cholesterol | 0.05 (-0.08, 0.19) | 0.426 | 0.01 (-0.07, 0.09) | 0.797 |
| Triglycerides** | 2.18 (-2.28, 6.65) | 0.336 | 0.92 (-1.57, 3.41) | 0.469 |
| Systolic blood pressure | -0.75 (-2.50, 1.01) | 0.403 | **-1.68 (-2.67, -0.68)** | **0.001*** |
| Diastolic blood pressure | 0.05 (-1.15, 1.26) | 0.930 | **-0.72 (-1.41, -0.03)** | **0.042** |
| Metabolic syndrome*** | 1.08 (0.76, 1.53) | 0.668 | 1.15 (0.94, 1.41) | 0.178 |
| Witnessed abuse |  |  |  |  |
| HbA1c** | - |  | -0.14 (-1.08, 0.81) | 0.776 |
| LDL cholesterol | - |  | 0.09 (0.00, 0.19) | 0.056 |
| HDL cholesterol | - |  | -0.02 (-0.06, 0.02) | 0.289 |
| Total cholesterol | - |  | 0.10 (-0.01, 0.21) | 0.090 |
| Triglycerides** | - |  | 2.61 (-1.14, 6.36) | 0.173 |
| Systolic blood pressure | - |  | -0.77 (-2.23, 0.70) | 0.305 |
| Diastolic blood pressure | - |  | 1.12 (0.83, 1.52) | 0.458 |
| Metabolic syndrome*** | - |  |  |  |
| Parental death |  |  |  |  |
| HbA1c** | 0.41 (-0.68, 1.50) | 0.460 | - |  |
| LDL cholesterol | 0.05 (-0.08, 0.18) | 0.444 | - |  |
| HDL cholesterol | -0.04 (-0.10, 0.01) | 0.098 | - |  |
| Total cholesterol | -0.04 (-0.18, 0.10) | 0.576 | - |  |
| Triglycerides** | -2.20 (-7.11, 2.70) | 0.377 | - |  |
| Systolic blood pressure | -0.58 (-2.49, 1.33) | 0.549 | - |  |
| Diastolic blood pressure | -0.04 (-1.38, 1.29) | 0.948 | - |  |
| Metabolic syndrome*** | 1.29 (0.90, 1.84) | 0.161 | - |  |
| Parental offending |  |  |  |  |
| HbA1c** | **1.45 (0.48, 2.43)** | **0.004*** | - |  |
| LDL cholesterol | -0.04 (-0.13, 0.06) | 0.435 | - |  |
| HDL cholesterol | -0.03 (-0.07, 0.01) | 0.177 | - |  |
| Total cholesterol | 0.03 (-0.07, 0.14) | 0.532 | - |  |
| Triglycerides** | **5.47 (1.88, 9.06)** | **0.003*** | - |  |
| Systolic blood pressure | 0.55 (-1.00, 2.09) | 0.488 | - |  |
| Diastolic blood pressure | 0.52 (-0.52, 1.56) | 0.326 | - |  |
| Metabolic syndrome*** | **1.28 (0.97, 1.70)** | **0.079** | - |  |
| Physical neglect |  |  |  |  |
| HbA1c** | 1.13 (-0.03, 2.30) | 0.056 | - |  |
| LDL cholesterol | -0.01 (-0.11, 0.10) | 0.904 | - |  |
| HDL cholesterol | **-0.07 (-0.11, -0.03)** | **0.002*** | - |  |
| Total cholesterol | 0.05 (-0.07, 0.16) | 0.449 | - |  |
| Triglycerides** | **7.10 (2.86, 11.34)** | **0.001*** | - |  |
| Systolic blood pressure | -0.56 (-2.21, 1.09) | 0.507 | - |  |
| Diastolic blood pressure | -0.33 (-1.43, 0.77) | 0.559 | - |  |
| Metabolic syndrome*** | 1.04 (0.77, 1.41) | 0.805 | - |  |
| Emotional neglect |  |  |  |  |
| HbA1c** | - |  | 0.22 (-0.42, 0.86) | 0.497 |
| LDL cholesterol | - |  | 0.02 (-0.05, 0.09) | 0.627 |
| HDL cholesterol | - |  | **-0.05 (-0.08, -0.02)** | **0.001*** |
| Total cholesterol | - |  | -0.01 (-0.09, 0.06) | 0.723 |
| Triglycerides** | - |  | 1.57 (-1.03, 4.16) | 0.236 |
| Systolic blood pressure | - |  | **-1.52 (-2.57, -0.48)** | **0.004*** |
| Diastolic blood pressure | - |  | -0.44 (-1.16, 0.28) | 0.226 |
| Metabolic syndrome*** | - |  | 1.17 (0.95, 1.44) | 0.128 |
| Physical abuse |  |  |  |  |
| HbA1c** | - |  | **1.04 (0.02, 2.07)** | **0.046** |
| LDL cholesterol | - |  | 0.10 (0.00, 0.19) | 0.060 |
| HDL cholesterol | - |  | -0.03 (-0.06, 0.01) | 0.204 |
| Total cholesterol | - |  | 0.09 (-0.03, 0.20) | 0.142 |
| Triglycerides** | - |  | 1.76 (-1.94, 5.45) | 0.351 |
| Systolic blood pressure | - |  | -0.65 (-2.09, 0.79) | 0.378 |
| Diastolic blood pressure | - |  | -0.28 (-1.27, 0.71) | 0.576 |
| Metabolic syndrome*** | - |  | 1.18 (0.89, 1.57) | 0.246 |
| Sexual abuse |  |  |  |  |
| HbA1c** | - |  | 0.38 (-1.41, 2.18) | 0.674 |
| LDL cholesterol | - |  | 0.11 (-0.08, 0.30) | 0.260 |
| HDL cholesterol | - |  | -0.02 (-0.10, 0.05) | 0.556 |
| Total cholesterol | - |  | 0.02 (-0.20, 0.23) | 0.860 |
| Triglycerides** | - |  | -3.81 (-11.18, 3.57) | 0.311 |
| Systolic blood pressure | - |  | -2.90 (-5.88, 0.08) | 0.056 |
| Diastolic blood pressure | - |  | -1.93 (-3.99, 0.13) | 0.066 |
| Metabolic syndrome*** | - |  | 0.91 (0.48, 1.73) | 0.767 |
| Psychological abuse |  |  |  |  |
| HbA1c** | - |  | 0.26 (-0.51, 1.03) | 0.507 |
| LDL cholesterol | - |  | 0.07 (-0.01, 0.15) | 0.082 |
| HDL cholesterol | - |  | **-0.05 (-0.08, -0.02)** | **0.003*** |
| Total cholesterol | - |  | 0.03 (-0.06, 0.12) | 0.533 |
| Triglycerides** | - |  | 1.19 (-1.76, 4.13) | 0.430 |
| Systolic blood pressure | - |  | -0.40 (-1.56, 0.76) | 0.498 |
| Diastolic blood pressure | - |  | 0.23 (-0.57, 1.02) | 0.574 |
| Metabolic syndrome*** | - |  | 1.18 (0.94, 1.48) | 0.151 |

*Note.* Associations with *p​≤*0.05 are presented in bold-face.

*Findings significant under Bonferroni correction (*p​≤*0.005).

**Results presented as % differences (95% CI) as outcomes were positively skewed and log transformed prior to analysis.

***Results presented as ORs (95% CI).

^a^ Models are adjusted for sex, father’s occupation at birth, maternal education level at birth, household overcrowding at 7 years, birth weight, gestational age, maternal age at birth, maternal smoking during pregnancy and mother’s BMI before childbirth. HbA1c analyses additionally adjust for antidiabetic medication; lipid analyses additionally adjust for lipid regulating medication; blood pressure analyses additionally adjust for antihypertensive medication; metabolic syndrome analyses additionally adjust for antidiabetic, lipid regulating and antihypertensive medication.

ACE, adverse childhood experience; BMI, body mass index; LDL, low-density lipoprotein; HDL, high-density lipoprotein; OR, odds ratio.

**Supplementary Table 6.** Regression coefficients (mean difference, % difference or OR) showing associations between ACE scores and cardiometabolic markers at age 44/45 in the NCDS (adjusting, not correcting, for those on treatment)

|  | Prospective childhood adversity | | | Retrospective childhood adversity | | |
| --- | --- | --- | --- | --- | --- | --- |
|  |  | *B* (95% CI)^a^ | *p* |  | *B* (95% CI)^a^ | *p* |
| HbA1c** |  |  |  |  |  |  |
|  | 0 ACEs | Ref |  | 0 ACEs | Ref |  |
|  | 1 ACEs | 0.49 (-0.12, 1.10) | 0.113 | 1 ACEs | -0.03 (-0.57, 0.52) | 0.926 |
|  | 2+ ACEs | 0.98 (-0.02, 1.98) | 0.055 | 2 ACEs | -0.37 (-1.04, 0.30) | 0.275 |
|  |  |  |  | 3 ACEs | -0.64 (-1.62, 0.35) | 0.203 |
|  |  |  |  | 4+ ACEs | 0.48 (-0.33, 1.29) | 0.248 |
| LDL cholesterol |  |  |  |  |  |  |
|  | 0 ACEs | Ref |  | 0 ACEs | Ref |  |
|  | 1 ACEs | **0.07 (0.00, 0.13)** | **0.039** | 1 ACEs | 0.01 (-0.05, 0.07) | 0.805 |
|  | 2+ ACEs | -0.01 (-0.10, 0.08) | 0.844 | 2 ACEs | 0.00 (-0.07, 0.08) | 0.993 |
|  |  |  |  | 3 ACEs | -0.06 (-0.16, 0.04) | 0.257 |
|  |  |  |  | 4+ ACEs | 0.05 (-0.03, 0.13) | 0.247 |
| HDL cholesterol |  |  |  |  |  |  |
|  | 0 ACEs | Ref |  | 0 ACEs | Ref |  |
|  | 1 ACEs | -0.02 (-0.05, 0.00) | 0.087 | 1 ACEs | -0.02 (-0.04, 0.01) | 0.200 |
|  | 2+ ACEs | **-0.06 (-0.10, -0.02)** | **0.003*** | 2 ACEs | 0.00 (-0.03, 0.03) | 0.956 |
|  |  |  |  | 3 ACEs | -0.02 (-0.07, 0.02) | 0.274 |
|  |  |  |  | 4+ ACEs | **-0.04 (-0.07, 0.00)** | **0.026** |
| Total cholesterol |  |  |  |  |  |  |
|  | 0 ACEs | Ref |  | 0 ACEs | Ref |  |
|  | 1 ACEs | **0.07 (0.00, 0.14)** | **0.047** | 1 ACEs | 0.01 (-0.05, 0.08) | 0.714 |
|  | 2+ ACEs | 0.02 (-0.09, 0.12) | 0.731 | 2 ACEs | 0.00 (-0.08, 0.09) | 0.951 |
|  |  |  |  | 3 ACEs | -0.10 (-0.21, 0.02) | 0.112 |
|  |  |  |  | 4+ ACEs | 0.02 (-0.08, 0.12) | 0.680 |
| Triglycerides** |  |  |  |  |  |  |
|  | 0 ACEs | Ref |  | 0 ACEs | Ref |  |
|  | 1 ACEs | 1.73 (-0.68, 4.14) | 0.158 | 1 ACEs | 1.28 (-1.04, 3.60) | 0.278 |
|  | 2+ ACEs | **4.42 (0.50, 8.34)** | **0.027** | 2 ACEs | 0.57 (-2.22, 3.36) | 0.688 |
|  |  |  |  | 3 ACEs | -1.04 (-4.97, 2.90) | 0.605 |
|  |  |  |  | 4+ ACEs | 1.13 (-1.94, 4.20) | 0.469 |
| Systolic blood pressure |  |  |  |  |  |  |
|  | 0 ACEs | Ref |  | 0 ACEs | Ref |  |
|  | 1 ACEs | -0.10 (-1.12, 0.91) | 0.839 | 1 ACEs | -0.01 (-0.93, 0.91) | 0.983 |
|  | 2+ ACEs | -0.09 (-1.57, 1.40) | 0.910 | 2 ACEs | -0.45 (-1.59, 0.69) | 0.441 |
|  |  |  |  | 3 ACEs | **-2.03 (-3.62, -0.45)** | **0.012** |
|  |  |  |  | 4+ ACEs | -0.75 (-2.01, 0.51) | 0.241 |
| Diastolic blood pressure |  |  |  |  |  |  |
|  | 0 ACEs | Ref |  | 0 ACEs | Ref |  |
|  | 1 ACEs | 0.06 (-0.63, 0.76) | 0.858 | 1 ACEs | 0.16 (-0.48, 0.79) | 0.631 |
|  | 2+ ACEs | 0.23 (-0.78, 1.25) | 0.648 | 2 ACEs | 0.10 (-0.68, 0.88) | 0.798 |
|  |  |  |  | 3 ACEs | -1.09 (-2.18, 0.00) | 0.051 |
|  |  |  |  | 4+ ACEs | -0.06 (-0.92, 0.81) | 0.898 |
| Metabolic syndrome*** |  |  |  |  |  |  |
|  | 0 ACEs | Ref |  | 0 ACEs | Ref |  |
|  | 1 ACEs | 1.00 (0.82, 1.23) | 0.963 | 1 ACEs | 1.15 (0.96, 1.38) | 0.137 |
|  | 2+ ACEs | 1.24 (0.95, 1.63) | 0.120 | 2 ACEs | 1.00 (0.79, 1.28) | 0.972 |
|  |  |  |  | 3 ACEs | 0.96 (0.69, 1.33) | 0.819 |
|  |  |  |  | 4+ ACEs | 1.20 (0.93, 1.56) | 0.161 |

*Note.* Associations with *p​≤*0.05 are presented in bold-face.

*Findings significant under Bonferroni correction (*p​≤*0.005).

**Results presented as % differences (95% CI) as outcomes were positively skewed and log transformed prior to analysis.

***Results presented as ORs (95% CI).

^a^ Models are adjusted for sex, father’s occupation at birth, maternal education level at birth, household overcrowding at 7 years, birth weight, gestational age, maternal age at birth, maternal smoking during pregnancy and mother’s BMI before childbirth. HbA1c analyses additionally adjust for antidiabetic medication; lipid analyses additionally adjust for lipid regulating medication; blood pressure analyses additionally adjust for antihypertensive medication; metabolic syndrome analyses additionally adjust for antidiabetic, lipid regulating and antihypertensive medication.

ACE, adverse childhood experience; BMI, body mass index; LDL, low-density lipoprotein; HDL, high-density lipoprotein; OR, odds ratio.
